# Advantages and pitfalls of an extended gene panel for investigating complex neurometabolic phenotypes

**DOI:** 10.1093/brain/aww221

**Published:** 2016-09-06

**Authors:** Emma S. Reid, Apostolos Papandreou, Suzanne Drury, Christopher Boustred, Wyatt W. Yue, Yehani Wedatilake, Clare Beesley, Thomas S. Jacques, Glenn Anderson, Lara Abulhoul, Alex Broomfield, Maureen Cleary, Stephanie Grunewald, Sophia M. Varadkar, Nick Lench, Shamima Rahman, Paul Gissen, Peter T. Clayton, Philippa B. Mills

**Affiliations:** aww221-1 1 Genetics and Genomics Medicine Programme, UCL Institute of Child Health, London, UK; aww221-2 2 Neurology Department, Great Ormond Street Hospital for Children NHS Foundation Trust, London, UK; aww221-3 3 North East Thames Regional Genetics Service, Great Ormond Street Hospital for Children NHS Foundation Trust, London, UK; aww221-4 4 Structural Genomics Consortium, University of Oxford, Oxford, UK; aww221-5 5 Histopathology Department, Great Ormond Street Hospital for Children NHS Foundation Trust, London, UK; aww221-6 6 Developmental Biology and Cancer Programme, UCL Institute of Child Health, London, UK; aww221-7 7 Metabolic Medicine Department, Great Ormond Street Hospital for Children NHS Foundation Trust, London, UK

**Keywords:** neurometabolic disorders, inborn errors of metabolism, heterogeneity, next-generation sequencing, gene panel

## Abstract

Neurometabolic disorders are markedly heterogeneous, both clinically and genetically, and are characterized by variable neurological dysfunction accompanied by suggestive neuroimaging or biochemical abnormalities. Despite early specialist input, delays in diagnosis and appropriate treatment initiation are common. Next-generation sequencing approaches still have limitations but are already enabling earlier and more efficient diagnoses in these patients. We designed a gene panel targeting 614 genes causing inborn errors of metabolism and tested its diagnostic efficacy in a paediatric cohort of 30 undiagnosed patients presenting with variable neurometabolic phenotypes. Genetic defects that could, at least partially, explain observed phenotypes were identified in 53% of cases. Where biochemical abnormalities pointing towards a particular gene defect were present, our panel identified diagnoses in 89% of patients. Phenotypes attributable to defects in more than one gene were seen in 13% of cases. The ability of *in silico* tools, including structure-guided prediction programmes to characterize novel missense variants were also interrogated. Our study expands the genetic, clinical and biochemical phenotypes of well-characterized (*POMGNT1*, *TPP1*) and recently identified disorders (*PGAP2*, *ACSF3*, *SERAC1*, *AFG3L2*, *DPYS*). Overall, our panel was accurate and efficient, demonstrating good potential for applying similar approaches to clinically and biochemically diverse neurometabolic disease cohorts.

## Teaser

Targeted gene panels can be used to establish molecular diagnoses in paediatric cohorts. Reid *et al*. report that this approach is accurate, efficient and can be preferred to whole-exome or genome sequencing for patients with neurological symptomatology and clues suggestive of an inherited metabolic disorder.

## Graphical abstract

**Figure 1g:**
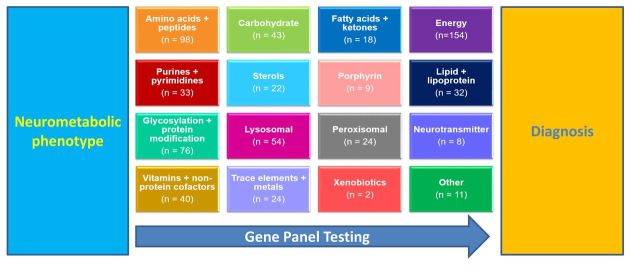
Targeted gene panels can be used to establish molecular diagnoses in paediatric cohorts. Reid *et al*. report that this approach is accurate, efficient and can be preferred to whole-exome or genome sequencing for patients with neurological symptomatology and clues suggestive of an inherited metabolic disorder.

## Introduction

Inborn errors of metabolism (IEM) are markedly heterogeneous, both clinically and genetically, with more than 600 genes known to cause disease. In the presence of neurological dysfunction, which is not only common in IEM but also often the most prominent phenotypic feature, these patients are frequently labelled as having ‘probable neurometabolic disease’, especially if suggestive neuroimaging or laboratory findings co-exist. The challenges when diagnosing neurometabolic disorders are largely attributable to the clinical and genetic heterogeneity (including often non-specific or atypical presentations early on in the disease course) and lack of clinical awareness of rare entities. Patients with suspected neurometabolic disease are frequently referred to specialist centres and undergo extensive and often invasive diagnostic testing. Despite this, diagnostic delays or difficulties establishing a definitive diagnosis are commonly encountered, with many such patients attending secondary and tertiary neurology clinics remaining undiagnosed (Verity *et al.*, 2010).

Timely diagnosis of neurometabolic disease is crucial, especially for those disorders that are treatable or manageable, with early initiation of treatment often resulting in improved outcomes. Next-generation sequencing (NGS) has revolutionized the diagnostic approach to such conditions (Nemeth *et al.*, 2013; Martin *et al.*, 2014) and helped to reduce the number of tests required for a diagnosis to be established. However, despite the continuous progress made in the field, there are still limitations to the approach, including access to NGS technology (especially in a non-specialist setting), costs, incomplete coverage of candidate genes and generation of large amounts of data that are difficult to interpret. Whole-exome sequencing (WES) and whole-genome sequencing (WGS) studies are primarily offered either in research laboratories or in a commercial setting, and have not yet been fully integrated into the clinical genetics services of many healthcare systems worldwide. An alternative NGS method, gene panel testing, has recently become available in clinical services and offers targeted testing of candidate genes. An extended genetic panel approach to investigating IEM might be advantageous (Saudi Mendeliome Group, 2015) due to reduced times required for data processing and increased coverage depth compared to WES and WGS. Our objective was to investigate the utility of this approach by designing an IEM gene panel and applying it to patients presenting with a wide array of neurometabolic phenotypes. We discuss the panel’s effectiveness in establishing a diagnosis, the clinical implications of its use as well as potential pitfalls of using broad-scale genetic testing. We also consider the predictive value of *in silico* tools commonly used for characterization of novel variants and investigate whether mapping of detected variants to known 3D protein structures can help further elucidate their significance.

## Materials and methods

### Patients

This study was approved by the National Research Ethics Service (NRES) Committee London – Bloomsbury (REC reference: 13/LO/0168). We recruited patients from a single UK tertiary centre’s neurometabolic disease clinics presenting with a range of neurological features such as developmental delay, macro or microcephaly, neurological regression, ataxia, epilepsy and/or organomegaly with or without other diagnostic indicators [including suggestive biochemical marker(s) or neuroimaging abnormalities]. All participants had undergone extensive previous investigations including multiple standard and specialized biochemical tests, invasive procedures (e.g. muscle and/or skin biopsy, lumbar puncture) and targeted gene testing but lacked a definitive molecular diagnosis. Thirty patients were included (Tables 1–3). First, we recruited 21 patients with suspected IEM but absence of specific clinical findings or biochemical pointers towards a particular disorder. Additionally, we included nine cases where biochemical findings indicated a particular disorder or group of disorders, not only to investigate the utility of this approach in more specific presentations but also because similar biochemical abnormalities could result from mutations in multiple genes. Finally, for panel validation purposes, we additionally recruited 13 patients with a known genetic diagnosis (Supplementary Table 1). Written informed consent was obtained in all cases.

**Table 1:** Clinical, biochemical and imaging phenotypes in patients who had pathogenic variants identified through gene panel sequencing

Patient	Age (years)	Gender	Primary clinical phenotype	Other phenotypic features	Relevant specialist investigations	MRI head	Diagnosis	Gene
B1	4	M	Macrocephaly, intermittent squint	Short stature, asthma, development unremarkable	Elevated 3-methylcrotonylglycine, methylcitrate and 3-hydroxyisovalerate in urine. Normal biotinidase activity	Not performed	Holocarboxylase synthetase deficiency	*HLCS*
B2	1	M	Sibling born prematurely and passed away due to hyper-ammonaemia. No clinical concerns	No	Moderately elevated orotic acid in urine between 9-48 µmol/mmol creatinine (ref: 0-5)	Not performed	Orotic aciduria	*UMPS*
B3	15	M	At 3 1/2 years: lethargy, vomiting, alkalosis and hyper-ammonaemia Learning and behavioural difficulties	Day 2 of life: to lethargy and irritability- presumed sepsis but cultures negative. NH_3_ not measured	Plasma aminoacids: raised glutamine. Low carbomylphosphate synthase activity in liver biopsy (0.15 mmol/hr/mg protein)	Not performed	Carbomoyl phosphate synthetase I deficiency	*CPS1*
B4	17	F	Short stature, obesity, distended abdomen at 3 years	Hepatomegaly. No documented learning difficulties	Low glycogen debranching enzyme activity of 0.07 µmol/min/g protein (ref: 0.3-3.0)	Not performed	Glycogen storage disease type III	*AGL*
B5	9	F	Galactosaemia, picked up through newborn screening, treated early	Normal development apart from difficulties in mathematics	Low gal-1-P-uridyltransferase activity Elevated galactose-1-phosphate	Not performed	Galactosaemia	*GALT*
B6	5	M	Global delay. One of monozygotic twins	Hypotonia, brachycephaly, long face	Elevated plasma lysine ranging between 439-449 µmol/l (ref: 100-300). Elevated CSF lysine of 67 µmol/l (ref: 10-32)	Delayed myelination, lack of white matter bulk	Hyperlysinaemia	*AASS*
B7	5	M	Global delay, epilepsy, one of monozygotic twins	Hypotonia, brachycephaly, long face	Elevated plasma lysine ranging between 440-780 µmol/l (ref: 100-300). Elevated CSF lysine of 92 µmol/l (ref: 10-32)	Not performed	Hyperlysinaemia	*AASS*
B8	20	F	Sensorineural hearing loss, ataxia, neurological regression	Scoliosis, constipation	Bile acid analysis and skin fibroblast studies suggestive of a peroxisomal biogenesis defect	Leukodystrophy	Peroxisome biogenesis disorder	*PEX6*
U1	11	F	Developmental delay, ataxia, horizontal nystagmus	Microcephaly, retinal dystrophy	CSF: low 5-MTHF and high HVA and BH4. Blood: Elevated prolactin, alanine, intermittently high CK and plasma lactate. Muscle: Normal RCE	Normal	Muscular dystrophy-dystroglycanopathy	*POMGNT1*
U2	2	M	Microcephaly, developmental delay	Dysplastic kidneys	Neonatal lactic acidosis, high plasma triglycerides, elevated urine thymidine and uracil, low plasma urate and detectable tymine	Normal	Dihydropyrimidinase deficiency	*DPYS*
U3	6	M	Neonatal jitteriness, developmental delay, autism	Joint hypermobility	Persistent methylmalonic and malonic aciduria	Not performed	Combined malonic and methylmalonic aciduria	*ACSF3*
U4	9	M	Congenital ataxia, diplegia; drop attacks, no obvious EEG correlate	No	Plasma: mildly raised alanine, normal lactate. CSF: low 5-HIAA	Abnormal bilateral caudate and lentiform neuclei signal	Spinocerebellar ataxia 28 / Autosomal recessive spastic ataxia 5	*AFG3L2*
U5	4	M	Developmental delay, subsequent regression with progressive dyskinetic movement disorder, dysphagia	Sensorineural deafness	Raised 3-methylglutaconic acid level with normal 3-methylglutaric levels	Basal ganglia high T_2_ signal, cerebellar atrophy	3-methylglutaconic aciduria with deafness, encephalopathy, and Leigh-like syndrome	*SERAC1*
U6	2	M	Global severe developmental delay, tonic seizures	Multiorgan malformations including VSD, Hirschprung’s. Dysmorphism	Recurrent hypoglycaemia; hypogammaglobulinaemia, hyperphosphatasia	Dandy-Walker malformation, reduced white matter bulk	Hyperphosphatasia with mental retardation syndrome 3	*PGAP2*
U7	6	M	Developmental delay, microcephaly, lower limb hyper-reflexia	No	Abnormal transferrin isoelectric focusing (type 1 pattern)	Lack of white matter bulk	Late infantile neuronal ceroid lipofuscinosis; Hereditary fructose intolerance	*TPP1 + ALDOB*

U8	6	M	Global developmental delay, Sensorineural hearing loss	Neonatal acute liver failure, resolved. Recurrent hypoglycaemia. Recurrent infections	Abnormal transferrin isoelectric focusing (type 1 pattern), normal phosphomannomutase and phosphomannisomerase. Low IgA/IgM, normal IgG and lymphocyte subsets	Normal	Galactose epimerase deficiency	*GALE*

5-HIAA = 5-hydroxyindoleacetic acid; 5-MTHF = 5-methyltetrahydrofolate; BH4 = tetrahydrobiopterin; CK = creatine kinase; F = female; HVA = homovanillic acid; M = male; RCE = respiratory chain enzymes; VSD = ventricular septal defect.

**Table 2:** Details of pathogenic variants identified in patients through gene panel sequencing

Patient	Gene	Nucleotide change	Amino acid	Segregation confirmed	SIFT	PolyPhen-2	CADD score	Reference	Clinical phenotype explained	Biochemical phenotype explained
B1	*HLCS*	c.2126C>T	p.Pro709Leu	No	Deleterious	Probably damaging	20.9	Novel	Yes	Yes
		c.1921G>A	p.Val641Met		Deleterious	Possibly damaging	24.8	Novel		
		c.1533dupT	p.Val512CysfsTer65		-	-	35.0	Novel		
B2	UMPS	c.451G>A	p.Val151Met	No	Deleterious	Probably damaging	26.7	Novel	Yes	Yes
B3	CPS1	c.1010A>G	p.His337Arg	No	Deleterious	Probably damaging	26.3	Aoshima *et al.* 2001	Yes^c^	Yes^c^
B4	AGL	c.2590C>T	p.Arg864Ter	No	-	-	38.0	Shen *et al.* 1996	Yes	Yes
		c.2590C>T	p.Arg864Ter							
B5	GALT	c.563A>G	p.Gln188Arg	No	Deleterious	Probably damaging	25.3	Reichardt *et al.* 1991	Yes	Yes
		c.584T>C	p.Leu195Pro		Deleterious	Benign	26.1	Reichardt *et al.* 1992		
B6	AASS	c.965G>A	p.Arg322His	No	Tolerated	Probably damaging	29.1	Novel	No	Yes
		c.965G>A	p.Arg322His							
B7	AASS	c.965G>A	p.Arg322His	No	Tolerated	Probably damaging	29.1	Novel	No	Yes
		c.965G>A	p.Arg322His							
B8	PEX6	c.2734G>A	p.Ala912Thr	No^a^	Deleterious	Probably damaging	34.0	Novel	Yes	Yes
		c.2734G>A	p.Ala912Thr							
U1	POMGNT1	c.373C>G	p.Arg125Gly	No	Tolerated	Benign	21.7	Novel	Yes	Yes
		c.1539+1G>A	-		-	-	28.1	Yoshida *et al.* 2001		
U2	DPYS	c.144_151dupGCTGCGGG	p.Val51GlyfsTer50	No	-	-	25.2		No	Yes
		c.144_151dupGCTGCGGG	p.Val51GlyfsTer50							
U3	ACSF3	c.1453A>C	p.Ser485Arg	Yes	Tolerated	Probably damaging	24.2	Novel	Yes	Yes
		c.1453A>C	p.Ser485Arg							
U4	AFG3L2	c.1067T>G	p.Leu356Arg	No	Deleterious	Probably damaging	29.9	Novel	Yes	Yes
		c.1067T>G	p.Leu356Arg							
U5	*SERAC1*	c.1850delinsCA	p.Ile617ThrfsTer6	Yes	-	-	35.0	Novel	Yes	Yes
		c.1850delinsCA	p.Ile617ThrfsTer6							
U6	*PGAP2*	c.560C>T	p.Ala187Val	Yes	Deleterious	Probably damaging	23.3	Novel	Yes	Yes
		c.560C>T	p.Ala187Val							
U7	*TPP1*	c.887G>A	p.Gly296Asp	No	Deleterious	Probably damaging	24.6	Novel	Yes	No
		c.887G>A	p.Gly296Asp							
	*ALDOB*	c.178C>T	p.Arg60Ter	No^b^	-	-	37.0	Santer *et al.*, 2005		Yes
		c.178C>T	p.Arg60Ter							
U8	*GALE*	c.280G>A	p.Val94Met	No	Deleterious	Possibly damaging	29.9	Wohlers *et al.*, 1999	Yes	Yes
		c.284G>A	p.Gly95Asp		Deleterious	Probably damaging	33.0	Novel		

The maximum minor allele frequency (MAF) for any variant considered to be potentially pathogenic was 0.5%.

^a^Parental DNA was unavailable but Sanger sequencing identified the same homozygous mutation in a similarly affected sister.

^b^Parental DNA was unavailable but Sanger sequencing identified the same homozygous mutation in a brother who also had an abnormal type I transferrin isoelectric focusing pattern.

^c^Second mutation not identified. CADD = Combined Annotation Dependent Depletion.

**Table 3:** Patients with no diagnosis identified though the genetic panel

ID	Gender	Age (years)	Primary neurological phenotype	Other phenotypic features	Relevant specialist investigations	MRI head	Eventual diagnosis
B9	F	6	Developmental delay, absence seizures	Bilateral sensorineural deafness	Grossly elevated plasma proline. Elevated n-pyrrole-2-carboxyglycine confirming hyperprolinaemia type II	Not performed	Hyperprolinaemia type II (novel homozygous deletion in *ALDH4A1*)
U9	F	7	Learning difficulties. Delayed motor milestones, reduced exercise tolerance, responsive to intra-muscular vitamin B12 injections	Joint hypermobility	Methylmalonic aciduria and high plasma homocysteine, muscle biopsy normal	Normal	Not yet reached
U10	M	8	Global developmental delay, neurological regression, dys phagia, epilepsy	Alopecia, reflux, neutropenia, platelet dysfunction	Intermittently elevated plasma lactate but normal CSF lactate, low plasma manganese	Leigh-like changes in the basal ganglia and brainstem	Not yet reached
U11	F	5	Episodes of severe ketotic hypoglycaemia with seizures	No	Normal acylcarnitines, plasma amino acids. Slightly low fructose-1,6-bisphosphatase activity	Not performed	Not yet reached
U12	F	3	Developmental delay and regression, dysphagia	No	Low vitamin B12	Delayed myelination	Multiple mutations, only one of which picked up by gene panel *CUBN* (p.Ala2194Val)
U13	F	5	One of similarly affected siblings. Parental consanguinity. Developmental delay, reduced exercise tolerance, joint hypermobility	Dysmorphic features. Pancreatic insufficiency and fat malabsorption	Several raised plasma amino acids. Muscle histology suggestive of mitochondrial disorder but RCE normal	Normal	Not yet reached
U14	F	8	One of similarly affected siblings. Parental consanguinity. Developmental delay, reduced exercise tolerance, joint hypermobility	Pancreatic insufficiency and fat malabsorption.	Several raised plasma amino acids.	Not performed	Not yet reached
U15	F	6	Global delay, microcephaly. Movement disorder with chorea and non-epileptic myoclonic jerks	Previous faltering growth. Renal tubular acidosis on NaHCO_3_ supplements	No other abnormalities	Delayed myelination	Not yet reached
U16	M	12	Global delay, seizures, dysphagia. Sibling with similar features	Dysmorphism	Persistent low arginine but normal lactate, carnitine profile and urine organic acids	Leukodystrophy	Unconfirmed *NDUFS1* deletions
U17	M	5	Global delay, retinal dystrophy, dystonic extensor spasms, epilepsy	Reflux, hip dislocation, scoliosis	EEG features consistent with Electrical Status Epilepticus in Sleep (ESES)	Progressive cerebral and cerebellar atrophy	Microarray: deletion at 7q36.2 - *de novo* change which includes *DPP6* gene, known to be associated with neurological disorders. DDD ongoing
U18	F	15	Developmental delay, paroxysmal episodes of gasping, opisthotonus and discomfort related to food ingestion	Distinct facial features. Abnormal maculae on OCT and slightly swollen optic discs	Abnormal visual evoked potential (VEP)/electroretinogram (ERG)	Non-progressive ventricular dilatation	*KANSL1* c.1635-3T>C; Koolen-de Vries syndrome. Diagnosis made by geneticists
U19	M	6	Global delay. 4-limb motor disorder with variable increased tone. Xp21 in-frame deletion within dystrophin gene	Sister with similar phenotype but without the dystrophin deletion	High CK. Very long chain fatty acids: moderately raised C26 and C26:C22 ratio.	Normal	Not yet reached
U20	M	1	Faltering growth, poor feeding, hypoglycaemia and lethargy at 5 months. Marked hypotonia and hyper-reflexia at presentation	Rapid evolution to multiorgan failure- passed away shortly afterwards	Lactic acidosis, persistent. NH_3_ 88 µmol/L. Mitochondrial genome analysis normal, *POLG* common mutations normal	Agenesis of corpus callosum and colpocephaly	*EARS2* variant picked up by the panel. Functional work underway to establish its significance.
U21	M	7	Global delay, acquired microcephaly	No	Persistently low levels of branched-chain amino acids in plasma and CSF	Lack of white matter bulk and delayed myelination	*BCKDK* mutations – gene not on panel. Diagnosis by targeted gene testing

After gene panel analysis, some of the patients in our cohort had diagnoses established via comparative genomic hybridization array or targeted genetic testing. Patient U29 had *EARS2* mutations identified through the panel which would be consistent with the clinical phenotype. However, as the same variants were also encountered in unrelated non-affected individuals, their significance is still being investigated through functional studies (data not shown). Patient U25 had *NDUFS1* deletions identified through our panel; however, these were not confirmed via Sanger sequencing. CK = creatine kinase; OCT = ocular computerized tomography; RCE = respiratory chain enzymes.

### Gene capture, sequencing and variant analysis

A custom HaloPlex target enrichment system (Agilent) was used to capture 614 genes, covering 16 broad classes of IEM (Supplementary material). Sequencing was performed using the HiSeq 2500 platform (Illumina). Sequence variants with putatively deleterious effects were confirmed by Sanger sequencing (Supplementary Table 4). To interrogate for potential pathogenicity in identified variants, we investigated whether variants had been reported previously as pathogenic, their frequency in the population, segregation within the family (where samples were available) and predicted functional impact utilizing SIFT (http://sift.bii.a-star.edu.sg/), PolyPhen-2 (http://genetics.bwh.harvard.edu/pph2/) and Combined Annotation Dependent Depletion (CADD) (http://cadd.gs.washington.edu/). Where possible, missense variants were mapped to known 3D protein structures and compared to *in silico* findings (Supplementary Table 5).

## Results

### Panel validation

Nineteen of 20 pathogenic sequence variants were identified in the 13 genetically diagnosed control samples (Supplementary Table 1). These included seven heterozygous and five homozygous missense, two heterozygous splice site mutations, a heterozygous single base insertion and four deletions ranging in size from 2 bp to ∼6 kbp. The homozygous 37-amino acid deletion in Patient D6 was not identified. Seven of 20 variants had not been previously reported in the literature.

### Clinical characteristics of undiagnosed cohort

Age ranged from 1 to 20 years (mean 7.2 years, median 6 years). Only 9/30 patients (Patients B1–B9, Tables 1–3) had abnormal biochemistry suggestive of an underlying genetic diagnosis, despite previous extensive testing in all cases. Our panel identified 21 variants in 16 patients, of which only seven had previously been reported in the literature (Reichardt *et al.*, 1991; 1992; Shen *et al.*, 1996; Wohlers *et al.*, 1999; Aoshima *et al.*, 2001; Yoshida *et al.*, 2001; Santer *et al.*, 2005). Ten variants were classified as pathogenic, 10 as likely pathogenic and one of uncertain significance (Richards *et al.*, 2015) (Supplementary Table 5). Variants included 15 missense, two nonsense, three insertions/deletions and one splice site mutation. Identified variants could at least partially explain the observed clinical phenotype in all cases.

Of nine patients with previous biochemical testing pointing towards a diagnosis, identification of pathogenic variants was possible for eight (88.8%). Parental DNA to check segregation within families was not available. We were unable to identify any potential pathogenic variants in Patient B9, whose biochemical profile suggested hyperprolinaemia type II and, in whom, a homozygous complex insertion/deletion event resulting in a frameshift and premature stop codon in *ALDH4A1* was subsequently identified via Sanger sequencing. Otherwise, in most other cases, two pathogenic variants were identified in each candidate gene.

We were also able to attain a molecular genetic diagnosis in 8/21 (38%) of patients without a biochemical marker pointing towards a specific genetic diagnosis (Tables 1–3). Two pathogenic (or likely pathogenic) variants were identified for each candidate gene. All variants were confirmed by Sanger sequencing in probands and family members where possible. Detailed clinical descriptions of these patients are given in the Supplementary material. In Patients B6, B7 and U2, the identified variants could explain the biochemical abnormalities but not other clinical features observed, indicating the presence of other, as yet unidentified gene defects. Additionally, Patient U7 had pathogenic variants identified in *ALDOB* and *TPP1*, while the clinical and biochemical phenotype was consistent with simultaneous presence of mutations in both genes (Supplementary material).

### 3D structure analysis

3D structural analysis of identified variants was performed using the ICM-Pro software (Molsoft LLC), when structural data were available for the proteins (Patients B2, U7 and U8) or for ‘close homologues/orthologues’ (Patients B8 and U4) (Supplementary Table 6). The impact of the amino acid substitution for six missense variants, all predicted to be deleterious and probably/possibly damaging by SIFT and PolyPhen-2, was determined by mapping them onto the wild-type structures and inspecting potential changes in bonding interactions, packing and secondary structures due to the amino acid substitution. In all cases, our structure-guided findings concurred with *in silico* prediction software, further supporting variant pathogenicity.

## Discussion

In our study, we investigated the utility of an extended gene panel in diagnosing patients with neurometabolic disorders. Due to the marked clinical, biochemical and genetic heterogeneity encountered in neurometabolic disease, targeted gene testing is often not advantageous, economical or efficient. The panel described in our study was shown to be a powerful tool that enhances the diagnostic ability in the clinical setting. It covers 614 genes, including the vast majority of genes currently known to cause neurometabolic disease, hence sharing similarities with WES approaches but with the added advantage of more optimal coverage of targeted areas (Kammermeier *et al.*, 2014). Indeed, coverage of targeted areas was similar or superior to that reported in other gene panels despite the large number of genes covered (Nemeth *et al.*, 2013; Yohe *et al.*, 2015). Moreover, the diagnosis rate in our study was comparable to, or higher than, that reported in similar approaches recently applied in other patient groups exhibiting phenotypic heterogeneity (Kammermeier *et al.*, 2014; Sommen *et al.*, 2016; Trump *et al.*, 2016).

We investigated patients with a wide array of, and often non-specific, neurometabolic symptomatology and were able to identify disease-causing mutations in a large number of cases. We interrogated 30 cases with no definitive molecular diagnosis despite having had all the pathology laboratory (including metabolic biochemistry) tests and imaging modalities that a tertiary referral metabolic centre considered might lead to a diagnosis. Of the 21/30 patients lacking pointers towards an underlying molecular diagnosis, pathogenic variants that explained all the clinical and biochemical findings were identified in seven (33%) and some of the phenotypic features in one (5%); demonstrating the effectiveness of this approach in a clinically heterogeneous, diagnostically challenging cohort. In these patients, there was no clear phenotypic or biochemical feature associated with higher or lower diagnostic rates on our panel, although study numbers preclude further conclusions. Additionally, where suggestive biochemical abnormalities existed, our panel efficiently led to a definitive genetic diagnosis in 8/9 cases. However, it is important to note that our cohort was recruited through a single tertiary referral centre, which may lead to selection bias. Therefore, further studies using large cohorts of patients consecutively enrolled from multiple metabolic medicine centres are warranted to establish the exact sensitivity and specificity of our panel. Nevertheless, we demonstrate that our extended panel approach, with subsequent focus on candidate gene(s), can be an initial relatively cost-effective approach to investigate patients with suspected neurometabolic disorders. Moreover, although applied to a paediatric cohort, our approach would arguably be even more useful in adult populations, where neurometabolic phenotypes can be even more atypical, presentations more variable and biochemical phenotypes even more subtle. Indeed, many lysosomal storage, mitochondrial, peroxisomal and other metabolic disorders present atypically in adults. For example, adrenoleukodystrophy can present as early-onset dementia (Kumar *et al.*, 1995). Patients with urea cycle disorders, organic acidaemias and Niemann Pick type C can also exhibit psychiatric manifestations (Sedel *et al.*, 2007). Thus, a comprehensive panel approach can have high utility in patients presenting with unexplained/atypical psychiatric or neurological manifestations.

Our study expands the genotypic and phenotypic spectrum of several disorders but also re-emphasizes the complexity of diagnosing patients with IEM. Patient U1 presented with a multi-system disorder and significant myopathy; however, due to unremarkable brain imaging and a non-diagnostic muscle biopsy (Supplementary Fig. 1), the diagnosis of *POMGNT1*-related dystroglycanopathy was delayed. Although uncommon, normal glycosylated α-dystroglycan immunofluorescence staining has been reported previously in *POMGNT1* patients (Clement *et al.*, 2008). Patient U7 had neurodevelopmental difficulties and hyper-reflexia, hence representing a mild *TPP1*-related phenotype compared to those typically reported in the literature (Breedveld *et al.*, 2004; Sun *et al.*, 2013), whereas his abnormal transferrin isoelectric focusing was attributable to the *ALDOB* mutations. Indeed, following variant identification, tripeptidyl peptidase I activity in patient leucocytes was found to be at the upper boundary of the affected range. The above cases demonstrate the spectrum of severity associated with IEM and how common it is for clinicians investigating neurometabolic disorders to be misguided by investigation results, with resulting diagnostic delays. For example, an abnormal transferrin pattern combined with neurological dysfunction would prompt investigations for congenital disorders of glycosylation (Scott *et al.*, 2014), which was the case in Patient U8 in whom variants in *GALE* were identified and UDP-galactose 4’-epimerase activity was subsequently found to be undetectable.

Apart from expanding the phenotypic spectrum of ‘well-described’ disorders, our results help expand the genotypic and phenotypic spectrum of recently described genetic conditions including *PGAP2* (Hansen *et al.*, 2013; Krawitz *et al.*, 2013), *ACSF3* (Sloan *et al.*, 2011), *DPYS* (van Kuilenburg *et al.*, 2010), *AFG3L2* (Pierson *et al.*, 2011) and *SERAC1* (Wortmann *et al.*, 2012). Hence, panel approaches enable clinicians to establish diagnoses in (and increase awareness of) ever broadening phenotypes and recently-described disorders, while at the same time circumventing problematic heterogeneity issues and potentially shortening the time to establish a definitive diagnosis for some patients.

Some patients with IEM have defects in more than one gene contributing to observed phenotypes. Patient U7 had mutations in *ALDOB* and *TPP1*. While mutations in *ALDOB* have been associated with abnormal transferrin patterns (Adamowicz *et al.*, 2007), the majority of clinical features seen in this case are likely attributable to the *TPP1* mutation (Breedveld *et al.*, 2004; Sun *et al.*, 2013). Similarly, Patients B6 and B7 had mutations in *AASS*, which would explain the hyperlysinaemia seen in both plasma and CSF but not the presence of developmental delay, microcephaly, hypotonia and epilepsy (Houten *et al.*, 2013). Patient U2 had mutations in *DPYS*, which are associated with abnormal purine and pyrimidine metabolites but not with dysplastic kidneys, eczema, microcephaly and developmental delay (van Kuilenburg *et al.*, 2010). The phenotypic features in these patients are most likely attributable to other, yet unidentified, genetic defects. The existence of pathogenic variants at two genetic loci in one patient is not surprising, as individuals have ∼3.5 million variants in their genome (Gonzaga-Jauregui *et al.*, 2012). A recent genetic study showed that 4.6% of participants had blended phenotypes resulting from two single gene defects (Yang *et al.*, 2014). The above issues further complicate the diagnosis of IEM and highlight the utility of NGS, especially in highly heterogeneous disorders while emphasizing the need for diagnosticians to perform elaborate clinical phenotyping and not over-rely on sequencing results, especially when identified gene defects do not account fully for the observed clinical picture.

Despite our panel’s usefulness, there were also limitations in our approach. No potential disease-causing gene alterations were identified in 14/30 patients. While established metrics indicate that our capture efficiency and depth of coverage was good overall (Supplementary Table 3), mutations may have been missed because of less efficient capture of GC-rich regions or low coverage due to sample complexity. It is also plausible that the disease-causing genes were not included in our design or that the causative mutations were intronic or within regulatory regions. We were also unable to identify the second pathogenic variant in Patient B3 (CPS1 deficiency), possibly because it lies within exon 21 (regions of which were only covered at a read depth of 3×), an intronic area or a promoter region. More research including WES or WGS in mutation-negative cases is warranted to reach further conclusions. Overall, our findings agree with previous studies indicating that, when analysed by NGS, targeted genetic regions can be inconsistently covered at read depths sufficient for comprehensive variant analysis (Dewey *et al.*, 2014). Additionally, although able to identify deletions, we were unable to detect the homozygous 111 bp deletion in Patient D6 or insertion/deletion event in Patient B9, which highlights the challenges of using NGS to detect copy number variants (Mullaney *et al.*, 2010). Indeed, some common pathogenic alleles can be missed by conventional sequencing approaches, including targeted NGS, unless methods are specifically adapted or additional assays are included to capture them. These can include deep intronic splice variants as in leukoencephalopathy with brainstem and spinal cord involvement and lactate elevation (van Berge *et al.*, 2014) or whole gene deletions and duplications as in Pelizaeus-Merzbacher disease (Lee *et al.*, 2006).

Finally, detection of variants of uncertain significance could pose a diagnostic and ethical issue, especially in patients with specific phenotypes where more targeted genetic testing could be a reasonable alternative. We firstly addressed this by following a ‘panel within a panel’ approach, initially interrogating genes in which mutations were likely to result in the observed phenotypes (e.g. *MUT*, *MCEE*, *ACSF3*, *ALDH6A1*, *MMAA*, *MMAB*, *SUCLA2*, *LMBRD1*, *ABCD4*, *MMADHC* and *MMACHC* in patients with methylmalonic aciduria) and expanding our search when no likely pathogenic variants were identified. Moreover, during the consenting process, we specifically counselled all study participants that they would not be informed about variants that were not deemed relevant to the clinical presentation. Utilizing expert phenotyping, current guidance on variant interpretation (Richards *et al.*, 2015) and close collaboration between clinicians and scientists interrogating the data is crucial for the above to be successfully implemented. Nevertheless, our study shows that such approaches are feasible, even in patients with more specific clinical and/or biochemical phenotypes. This approach is particularly applicable in various neurometabolic conditions (such as the cases of peroxisomal biogenesis disorders and congenital disorders of glycosylation in our cohort), where mutations in a large number of genes could lead to similar biochemical abnormalities.

We also encountered difficulties when utilizing *in silico* tools for novel missense variant interpretation. When using SIFT and PolyPhen-2 interpretation, discordance was occasionally evident, not only for novel variants but also for common variants of established pathogenicity in *ASL* (Linnebank *et al.*, 2002) and *GALT* (Reichardt *et al.*, 1992) (Tables 2 and Supplementary Table 1). However, despite this discordance, CADD scores for these variants rank them more deleterious than 99.5% of all possible human single nucleotide variants. Additionally, SIFT, PolyPhen-2 and CADD suggested that a known pathogenic *IDUA* variant (Bach *et al.*, 1993) was not likely to be deleterious (Supplementary Table 1). Inability of online prediction tools, particularly those using sequence-based algorithms, to predict pathogenicity of all variants analysed correctly has been evaluated previously (Castellana and Mazza, 2013; Dong *et al.*, 2015; Walters-Sen *et al.*, 2015). *In silico* tools remain invaluable in filtering large numbers of variants identified using NGS platforms; however, further evidence to support or refute pathogenicity should be sought (Richards *et al.*, 2015), for example segregation analysis and enzymatic assays in appropriate patient tissues. In our study, we further characterized identified missense variants by mapping them to 3D protein structures where possible. All variants were predicted to be deleterious and probably/possibly damaging by SIFT and PolyPhen-2 and structural analysis supported these predictions in all cases, providing further evidence of pathogenicity. Should 3D structural information become available for larger parts of the human exome, this approach could become a valuable aid towards novel variant analysis (Yue *et al.*, 2014).

Extended panel approaches have gained popularity and are used by many clinical laboratories in the investigation of a wide range of genetically heterogeneous conditions (http://www.labs.gosh.nhs.uk/media/759058/goshome_v7.pdf) including neurometabolic disease. With decreasing NGS costs and the advent of the Genomics England 100 000 Genomes Project, WES and WGS will likely supersede the use of gene panels in the clinical diagnostic setting in the future. However, many challenges remain prior to this implementation, including difficulties in interpreting overwhelming amounts of data generated and uncertainties about clinically reportable findings (Dewey *et al.*, 2014). Moreover, WES and WGS have proven invaluable in the identification of novel genes (Saitsu *et al.*, 2013; Howard *et al.*, 2014) but such findings are not currently actionable within the diagnostic setting. Elucidating the significance of these variants is not possible without functional characterization in appropriate settings and models, which is often expensive and beyond the capacity of most clinical diagnostic laboratories. Until such challenges are surpassed, gene panel approaches provide a rapid and cost-effective method of testing patients with neurometabolic disorders and enable more timely diagnosis and prompt treatment initiation in these conditions.

## Funding

P.B.M. and P.T.C. received Great Ormond Street Hospital Children’s Charity (GOSHCC) Leadership awards (V2516 and V1254). P.G. is a Wellcome Trust Senior Research fellow. S.R. is supported by Great Ormond Street Hospital Children's Charity (GOSHCC) Research Leadership Grant V1260. A.P. holds a joint Action Medical Research (AMR) and British Paediatric Neurology Association (BPNA) fellowship. This project was funded by grants from the University College London Impact Award and GOSHCC Metabolic Fund and supported by the National Institute for Health Research Biomedical Research Centre at Great Ormond Street Hospital for Children NHS Foundation Trust and University College London.

## Supplementary material

Supplementary material is available here.

## Supplementary Material

Supplementary Data

## Abbreviations

IEM: inborn errors of metabolism
NGS: next-generation sequencing
WES: whole-exome sequencing
WGS: whole-genome sequencing
