# Supplementary material for: Advantages and pitfalls of an extended gene panel for investigating complex neurometabolic phenotypes
Source: Brain. 2016 Sep 6;139(11):2844–54. doi: 10.1093/brain/aww221 (PMC5091046; doi:10.1093/brain/aww221)
Supplement: Supplementary Data [file aww221_supplementary_data.zip › brain-2016-00692-File012.pdf]

METHODS

Targeted capture using HaloPlex target enrichment and library sequencing

A total of 614 genes known to cause IEM were targeted for capture and sequencing **(Supplementary File 1)**. These cover 16 broad classes of IEM **(Supplementary Table 2)**. The design was based on the Society for the Study of Inborn Errors of Metabolism (SSIEM) Classification of Inborn Errors of Metabolism (2011) (www.ssiem.org) with additions from recent publications and existing smaller disease-specific panels (Mitome200-Nuclear [Baylor College of Medicine] and Mitochondrial Disorders Panel [ARUP Laboratories]).

Disorder Class	Number of genes
**Disorders of amino acid and peptide metabolism**	98
**Disorders of carbohydrate metabolism**	43
**Disorders of fatty acid and ketone body metabolism**	18
**Disorders of energy metabolism**	154
**Disorders in the metabolism of purines, pyrimidines and nucleotides**	33
**Disorders in the metabolism of sterols**	22
**Disorders of porphyrin and haem metabolism**	9
**Disorders of lipid and lipoprotein metabolism**	32
**Congenital disorders of glycosylation and other disorders of protein modification**	76
**Lysosomal disorders**	54
**Peroxisomal disorders**	24
**Disorders of neurotransmitter metabolism**	8
**Disorders in the metabolism of vitamins and (non-protein) cofactors**	40
**Disorders in the metabolism of trace elements and metals**	24
**Disorders and variants in the metabolism of xenobiotics**	2
**Other disorders**	11

Supplementary Table 2: List of 16 classes of IEM and the distribution of the 614 genes amongst them. Note that due to the highly interconnected and heterogeneous nature of metabolism, mutations in one gene can give rise to multiple different disorders, leading to an apparently higher total number of genes than stated in the text.

The gene accession numbers and coordinates were downloaded from the UCSC Genome Browser (www.genome.ucsc.edu) and the SureDesign system (Agilent Technologies Inc. USA) was used to design the probe library for the genes of interest. Probes were designed to allow amplification of exons with 25bp of the flanking intronic sequence, selecting amplicons from both strands and optimised to ensure that all haplotype combinations of regions that were initially predicted to be poorly covered were amplified. A total of 7522 regions from the 614

genes were targeted with a final capture size of 1.43 Mbp. Genomic DNA was extracted from EDTA blood using the AutoGenFlex STAR automated system (Autogen, Holliston, MA) according to the manufacturer's instructions. Targeted sequence enrichment was performed using the Agilent HaloPlex Target Enrichment Kit (Agilent Technologies Inc. USA) in batches of 12 samples according to the manufacturer's instructions. Sample quality was verified at two time points during the protocol using an Agilent 2100 BioAnalyzer (Agilent Technologies, Wokingham, UK). Sample-specific barcodes were incorporated and DNA libraries were quantified using a Qubit 2.0 fluorometer. Samples were pooled in equimolar concentrations and spiked with PhiX virus library to serve as an internal control. Twenty five samples were sequenced per flow cell on the Illumina HiSeq 2500 platform using 150bp paired-end chemistry.

Variant calling, annotation and filtering and coverage analysis

Sequencing reads were imported from FASTQ files and HaloPlex adaptors were trimmed using Cutadapt v1.3. Reads were aligned to hg19 using the Burrows-Wheeler Aligner (BWA) software. Variants were called using VarScan v2.3 according to a minimum of 30X coverage with five alternate reads, a Phred base quality of 20 and without strand bias. CNVs on the processed BAM files were called using an in-house read depth pipeline. Variants were annotated based on Ensembl gene transcripts. Variants listed in the 1000 genomes database (http://browser.1000genomes.org/index.html) or Ensembl (African, American or European population frequencies) (http://www.ensembl.org) with a minor allele frequency (MAF) of greater than 2% were filtered out. Prioritisation of variants was carried out based on the following criteria: selection of candidate genes based on patient's phenotype, predicted effect on gene function, conservation of amino acid position, and frequency of the variant in the literature and databases including the 1000 genomes database, Ensembl and the Human Gene Mutation Database (HGMD http://www.biobase-international.com). Selection of candidate genes based on patient's phenotype was carried out using Phenomizer (http://compbio.charite.de/phenomizer/), a resource based on the Human Phenotype Ontology, or by filtering by disease class where appropriate.

Coverage analysis

Data on parts of exons and genes with a minimum of 30X coverage were generated automatically; however, further information could be manually extracted as required, for

example in cases of suggestive clinical phenotypes with identification of only one pathogenic variant in the candidate gene.

Sequencing metrics

The gene panel was optimised to target 7522 exons +/- 25 bps of the 614 genes of interest. This created a 1.43Mbp design with predicted target coverage of 99.55%. A mean depth of sequencing coverage 924.5X was observed and 97.2% of the targeted regions were successfully sequenced with a depth of coverage >20X. On average, 1562 (range: 1456 – 1658) and 28 (range: 14 – 38) variants were identified per patient before and after basic filtering, respectively. This filtering involved discarding variants identified in more than one patient (excluding other family members), variants with a minor allele frequency > 2% and intronic variants. The analysis of two affected siblings increased the analysis power and helped reduce candidate variants.

	Mean	Range
**Alignment (%)**	44.6	43.2 – 45.4
**ROI coverage > 10X (%)**	98.3	98.0 – 98.5
**ROI coverage > 20X (%)**	97.8	97.4 – 98.1
**ROI coverage > 30X (%)**	97.2	96.8 – 97.7
**ROI coverage > 50X (%)**	96.2	95.6 – 97.0
**ROI coverage > 100X (%)**	93.4	92.3 – 94.9
**Mean base coverage per sample**	987.8	969.6 – 1006.0

Supplementary Table 3: Percentage and range of alignment for all samples sequenced on the Illumina HiSeq platform in the regions of interest (ROI). The mean percentage and range of exons covered at greater than 10X, 20X, 30X, 50X and 100X are also shown. Finally, the mean and range of base coverage across the samples are shown.

Variant confirmation and segregation analysis

Sequence variants with putatively deleterious effects were confirmed by conventional bidirectional Sanger sequencing using standard protocols on an ABI DNA Sequencer (Life Technologies, Paisley, UK). Primer sequences used for the validations are as detailed in **Supplementary Table 4**. PCR products were used as templates for sequencing with BigDye version 1.1. Sequence data was aligned to the reference sequence (NCBI build 37) and analysed using Sequencher 4.10.1 software (Gene Codes, Ann Arbor, MI).

Gene (direction)	Sequence (5’ → 3’)	T_m (°C)
***GALE* (F)**	GCATTGCCAAGGACTAAAACC	62
***GALE* (R)**	CTAGTGTCTGTGCCCTGTCC	62
***POMGNT1* splice (F)**	GTCCATGTCTGCCAGCTCT	64
***POMGNT1* splice (R)**	CCCAAGGTTACATGGCTAGC	64
***POMGNT1* missense (F)**	TGTTTCAAGCAGCTGGTGTT	58
***POMGNT1* missense (R)**	ACTTCTGGTGAGTTGGTGTCA	58
***ACSF3* (F)**	CTGGCATAGCTGTTTCTCCG	62
***ACSF3* (R)**	GACTCATCTGCAGTCGTCTAA	62
***PEX6* (F)**	TGCCAACTCTGTTTCTTCCTG	60
***PEX6* (R)**	CCTCAAACTCCTGGGCTCAA	60
***AFG3L2* (F)**	TGTTCTACCATAGCTCAGATGTT	60
***AFG3L2* (R)**	AGGGCCATCTCTAGCAAGTG	60
***SERAC1* (F)**	CCCATTCGGCCTCTTTCAGT	62
***SERAC1* (R)**	TACAGCGCTTGAAGGGAGAA	62
***PGAP2* (F)**	GAGGACTTCAGTGGGTGCC	60
***PGAP2* (R)**	TTTTCTTCTGGGCTGCCTTG	60
***DPYS* (F)**	TCCGGATTTGCAGCCTGA	64
***DPYS* (R)**	GACCCCAGCGAAGAGAATCT	64
ALDOB* (F)	GGTCTTCTCCCTGGAACAC	62
ALDOB* (R)	GATGGAAAAGGGTGAGAAGAGA	62

Supplementary Table 4: Primer sequences used for validation of gene panel findings. Polymerase chain reaction was carried out in a final volumes of 20 µL using standard reagents (Bioline, London, UK): 15.55 µL dH2O, 2.5 µL 10X PCR reaction buffer, 0.75 µL 25 mM MgCl2, 2.5 µL 10 mM dNTPs, 1.25 µL forward and reverse primer, 0.2 µL Taq DNA polymerase, 1 µL genomic DNA. Cycling conditions were 96°C for 5 minutes, followed by 35 cycles of 30 seconds at 96°C, 30 seconds at T_m°C, 30 seconds at 72°C, and a final extension at 72°C for 10 minutes. * Primers to amplify *TPP1* are not listed because confirmatory Sanger sequencing was performed on a clinical basis at North East Thames Regional Genetic Service, GOSH, UK.

In order to identify the potential pathogenicity in identified variants, we investigated whether the variant had been reported as pathogenic previously, its frequency in the population, segregation of the variant within the family (where samples were available) and predicted functional impact utilising *in silico* tools. SIFT (http://sift.bii.a-star.edu.sg/) and PolyPhen-2 (http://genetics.bwh.harvard.edu/pph2/) were used to predict the pathogenicity of non-synonymous variants.

Variant Classification according to latest guidance

Classification of the variants identified in the 30 patients without a prior genetic diagnosis according to the recommendations of the American College of Medical Genetics and Genomics (AMCG) guidelines (Richards *et al.*, 2015).

Gene	Variant	Very Strong Evidence	Strong Evidence	Moderate Evidence	Supporting evidence	Final Classification
HLCS	c.2126C>T; p.Pro709Leu			PM2	PP2, PP3, PP4	Uncertain significance
HLCS	c.1921G>A; p.Val641Met			PM1, PM2	PP2, PP3, PP4	Likely pathogenic
HLCS	c.1533dupT; p.Val512CysfsTer65	PVS1		PM2	PP2, PP4	Pathogenic
UMPS	c.451G>A; p.Val151Met			PM1, PM2	PP2, PP3, PP4	Likely pathogenic
CPS1	c.1010A>G; p.His337Arg		PS1, PS3	PM1, PM2	PP2, PP3, PP4	Pathogenic
AGL	c.2590C>T; p.Arg864Ter		PS1, PS3	PM2	PP2, PP4	Pathogenic
GALT	c.563A>G; p.Gln188Arg		PS1, PS3	PM1, PM2, PM3	PP2, PP3, PP4	Pathogenic
GALT	c.584T>C; p.Leu195Pro		PS1, PS3	PM1, PM2, PM3	PP2, PP3, PP4	Pathogenic
AASS	c.965G>A; p.Arg322His			PM2	PP1, PP2, PP3, PP4	Likely pathogenic
POMGNT1	c.373C>G; p.Arg125Gly			PM2, PM3	PP2, PP3, PP4	Likely pathogenic
POMGNT1	c.1539+1G>A; -	PVS1	PS3	PM2	PP2, PP4	Pathogenic
DPYS	c.144_151dupGCTGCGGG; p.Val51GlyTer50	PVS1		PM2	PP2, PP4	Pathogenic
ACSF3	c.1453A>C; p.Ser485Arg			PM1, PM2	PP1, PP2, PP3, PP4	Likely pathogenic
PEX6	c.2734G>A; p.Ala912Thr			PM1, PM2	PP1, PP2, PP3, PP4	Likely pathogenic
AFG3L2	c.1067T>G; p.Leu356Arg			PM1, PM2	PP1, PP2, PP3, PP4	Likely pathogenic
SERAC1	c.1850_1851delinsCA; p.Ile617ThrfsTer6	PVS1		PM2	PP1, PP2, PP4	Pathogenic
PGAP2	c.560C>T; p.Ala187Val			PM2	PP1, , PP2, PP3, PP4	Likely pathogenic
TPP1	c.887G>A; p.Gly296Asp			PM1, PM2	PP2, PP3, PP4	Likely pathogenic
ALDOB	c.178C>T; p.Arg60Ter	PVS1	PS3	PM2	PP1, PP2, PP3, PP4	Pathogenic
GALE	c.280G>A; p.Val94Met		PS1, PS3	PM1, PM2	PP2, PP3, PP4	Pathogenic
GALE	c.284G>A; p.Gly95Asp			PM1, PM2, PM3	PP2, PP3, PP4	Likely pathogenic

Supplementary Table 5: Variant Classification in the undiagnosed cohort. Variants identified in patients without a prior genetic diagnosis were classified as pathogenic, likely pathogenic (> 90% certainty of being disease-causing) or of uncertain significance according to the recommendations of the American College of Medical Genetics and Genomics (ACMG) on variant interpretation (Richards *et al.*, 2015). PVS1: null variant (nonsense, frameshift, canonical +/- 1 or 2 splice sites, initiation codon, single or multi-exon deletion) in a gene where loss of function is a known mechanism of disease; PS1: same amino acid change as a previously

established pathogenic variant regardless of nucleotide change; PS3: well-established *in vitro* or *in vivo* functional studies supportive of a damaging effect on the gene or gene product; PM1: located in a mutational hot spot and/or critical and well-established functional domain without benign variation; PM2: Absent from controls (or at extremely low frequency if recessive) in Exome Sequencing Project, 1000 Genomes Project or Exome Aggregation Consortium; PM3: for recessive disorders, detected in *trans* with a pathogenic variant; PP1: co-segregation with disease in multiple affected family members in a gene definitively known to cause disease; PP2: missense variant in a gene that has a low rate of benign missense variation and in which missense variants are a common mechanism of disease; PP3: multiple lines of computational evidence support a deleterious effect on the gene or gene product (conservation, evolutionary, splicing impact, etc.); PP4: patient's phenotype or family history is high specific for a disease with a single genetic aetiology.

Identified Variant 3D mapping and Protein Structural Analysis

Structures of several human metabolic enzymes from diverse protein families have been deposited in the public domain within the Protein Database (PDB). We used these structures where available, alongside those of prokaryotic/eukaryotic homologues, to investigate missense variants identified and compare results with SIFT and Polyphen-2 findings. We 'modelled' the missense variation onto the 3D wild-type conformation of the protein and visually inspected the atomic environment surrounding the mutation. Thus, we assessed of the effects of the identified variants on interactions of the substrate or necessary cofactors at the active site, dimerization of the protein, or structural features such as di-sulphide bridges.

REFERENCES

Richards S, Aziz N, Bale S, Bick D, Das S, Gastier-Foster J, *et al.* Standards and guidelines for the interpretation of sequence variants: a joint consensus recommendation of the American College of Medical Genetics and Genomics and the Association for Molecular Pathology. Genet Med 2015; 17(5): 405-24.
