# Supplementary material for: Advantages and pitfalls of an extended gene panel for investigating complex neurometabolic phenotypes
Source: Brain. 2016 Sep 6;139(11):2844–54. doi: 10.1093/brain/aww221 (PMC5091046; doi:10.1093/brain/aww221)
Supplement: Supplementary Data [file aww221_supplementary_data.zip › brain-2016-00692-File011.pdf]

ADDITIONAL CLINICAL INFORMATION

Patient U1 (*POMGNT1*)

Age and gender: 11 years, female.

Relevant family history: N/A

Clinical presentation: Born by normal vaginal delivery at 36 weeks gestation. Admitted to hospital at 8 weeks with pneumonia alongside hypotonia, abnormal movements and required nasogastric tube feeding. Main presenting features at 4 years were poor appetite, developmental delay, frequent falls, limited exercise tolerance, ataxia, horizontal nystagmus, left strabismus and developmental delay (could only speak two words and indicated needs by pointing). Appearance is coarse with hirsutism. She can walk and run although is ataxic.

Biochemical/metabolic findings: Elevated plasma and CSF lactate (7.0 and 2.7 nmol/L) and intermittently raised creatine kinase (CK) at 8 weeks and 4 years of age. Normal chromosomal analysis, white cell ubiquinone, muscle respiratory chain enzymology and histology, fatty acid oxidation and pyruvate dehydrogenase in fibroblasts. CSF neurotransmitters showed low 5-methyltetrahydrofolate.

Brain imaging: Normal brain MRI at 7 years of age.

Treatment: Calcium folinate therapy and vitamin supplements indicated for a possible mitochondrial disorder (these were found to have no effect so were ceased).

Diagnosis and further investigations: Identified compound heterozygous mutations in the *POMGNT1* gene. The first is a splice variant (c.1539+1G>A) affecting the invariant GT donor splice site, which has been reported previously and has been shown to generate two aberrant mRNA transripts; a read-through of intron 17 with a premature stop codon at position 484 and a skipping of exon 17 resulting in an in-frame deletion of 42 amino acids (Yoshida *et al.*, 2001). The second is a novel missense variant (p.Arg125Gly) which is predicted to be tolerated and benign by SIFT and PolyPhen-2, respectively. Whilst the presence of *POMGNT1* has not been reported in lower organisms, an alignment of *POMGNT1* from higher organisms shows that Arg125 is conserved from humans to zebrafish. Upon identification of these two *POMGNT1* variants, a muscle biopsy taken at seven years of age was re-evaluated. There was increased variation in fibre size ranging from 7 – 37 µm (normal: 31 – 39 µm) due to scattered small angular and rounded fibres, as well as prominent lipid for the age of the patient. Subsequent

diagnostic immunohistochemistry and immunofluorescence staining of glycosylated α-dystroglycan using the VIA4 antibody appeared normal (**Supplementary Figure 1**) with no loss of α-dystroglycan being reported. This is consistent with the mild end of clinical spectrum of POMGNT1-related disease.

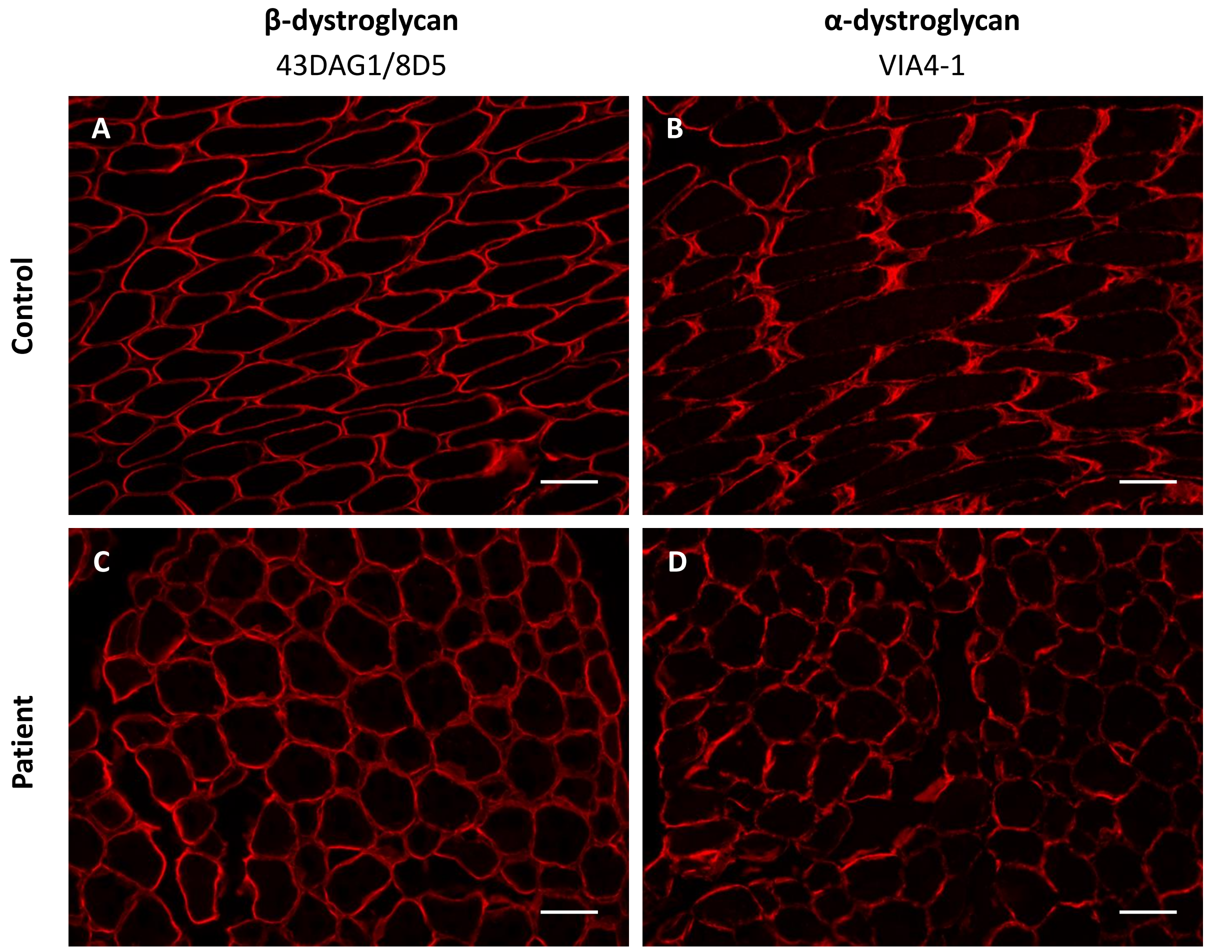

Supplementary Figure 1: Muscle biopsy analysis in patient U8. A and C; Immunofluorescence staining for β-dystroglycan of skeletal muscle from healthy control and patient. B and D; Immunofluorescence for α-dystroglycan using antibody against the glycosylated epitope (VIA4-1 antibody). Scale bars: 50 µm.

<u>Patient U2 (*DPYS*)</u>

Age and gender: 2 years, male.

Relevant family history: Previous male sibling death due to renal failure presenting with dysplastic kidneys, anaemia and raised triglycerides.

Clinical presentation: Antenatal diagnosis of dysplastic kidneys and anhydramnios. Intubated and ventilated at birth with deteriorating renal function which normalised in the following days. Presenting features are eczema, microcephaly, developmental delay, short stature, palpable liver and small kidneys with normal function.

Biochemical/metabolic findings: Transient neonatal lactic acidosis (up to 8.4 nmol/L, now within normal range), high plasma triglycerides (3.73 mmol/L) and elevated urine thymidine (220 µmol/L) and uracil (850 µmol/L) with an associated plasma low urate (86 µmol/L) and detectable thymine (1.8 µmol/L) suggestive of dihydropyrimidine dehydrogenase deficiency.

Brain imaging: Normal.

Treatment: Unknown.

Diagnosis and further investigations: Identified a novel homozygous duplication of a short sequence [ACCCGCAGC] within exon 1 of the DPYS gene which is predicted to cause a frameshift and a premature stop codon (p.Val51GlyfsTer50) producing a truncated protein that is likely to undergo nonsense-mediated decay. Mutations in this gene cause dihydropyrimidinuria, of which less than a dozen affected individuals have been reported. This mutation offers an explanation for aspects of the patient's biochemical phenotype, namely the elevation of thymidine and uracil. However, no other candidate mutations were found to explain the remaining features such as the dysplastic kidneys, eczema, microcephaly and developmental delay.

Patient U3 (*ACSF3*)

Age and gender: 6 years, male.

Relevant family history: Third child born to consanguineous parents. One previous still birth and two cousins living abroad affected by seizures and developmental delay.

Clinical presentation: Self-resolving neonatal jitteriness and laryngomalacia. Delayed motor milestones in the first year of life. Autism and speech and language delay.

Biochemical/metabolic findings: Urinary MMA/creatinine ratio: 36 – 89 µmol/mmol on two occasions over the course of four years (ref: 0 – 30) with normal methylcitrate levels, plasma homocysteine: 4 µmol/L (ref: 5 – 15), plasma MMA: 2.39 µmol/L, mild generalised

aminoaciduria, urinary N-acetyl-beta-D-glucosaminidase: 76 unit/µmol creatinine. Normal methylcitrate/creatinine ratio, vitamin B_{12}, blood spot carnitine profile and CSF amino acids.

Brain imaging: Normal.

Treatment: Unknown.

Diagnosis and further investigations: Identified a novel homozygous missense mutation (p.Ser485Arg) in *ACSF3* which has been shown to segregate within the family, both parents being heterozygous for this variant. This variant is predicted to be either "tolerated" or "probably damaging" by SIFT and PolyPhen-2, respectively. Following the identification of this mutation, urinary organic acid results were scrutinised and an intermittent mild elevation of malonic acid was detected. The constellation of clinical features combined with the malonic and methylmalonic aciduria would be consistent with an *ACSF3* defect.

Patient U4 (*AFG3L2*)

Age and gender: 9 years, male.

Relevant family history: Second child born to first cousin Pakistani parents.

Clinical presentation: Delayed walking (18 months) with frequent falls and delayed speech (at 4 ½ years had short phrases in parent's language of Punjabi/Urdu but very little English). He suffers frequent drop attacks consisting of episodes of acute onset marked hypotonia associated with noisy breathing and cyanosis. Examination at 9 years revealed generalised dystonia with increased lower limb tone, sustained ankle clonus and generalised brisk reflexes, poor fine motor skills, uncoordinated voluntary tongue movements, an inability to blow up his cheeks and difficulty in following with his eyes. Major clinical features were deemed to be ataxia with diplegia, developmental delay and drop attacks.

Biochemical/metabolic findings: Normal CSF lactate, neurotransmitter analysis, white cell enzymes, pyruvate dehydrogenase and muscle mitochondrial respiratory chain enzymes. Muscle ultrastructural analysis revealed structurally normal muscle architecture with a small proportion of mitochondria having a disorganised cristae structure, usually associated with close proximity to large lipid droplets (**Supplementary Figure 2**).

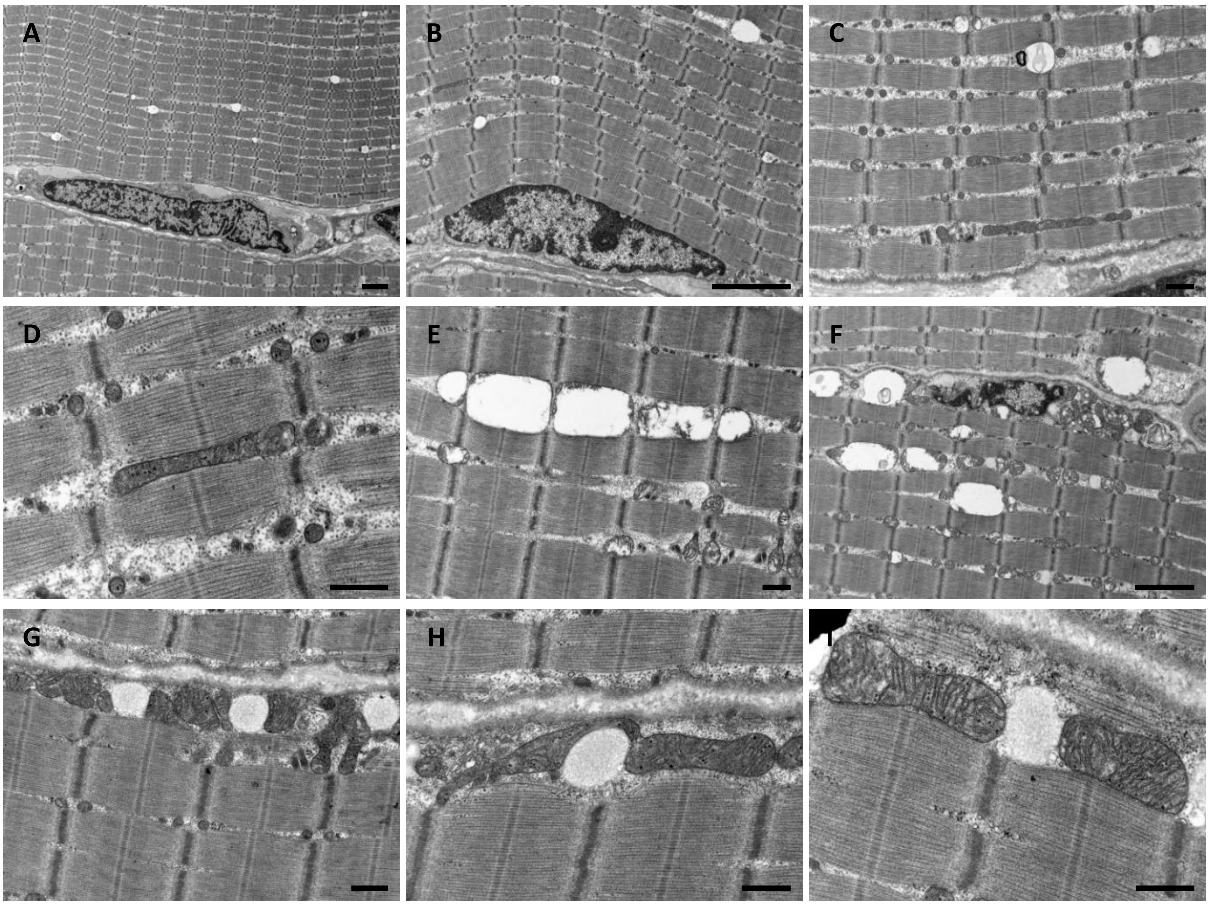

Supplementary Figure 2: Electron microscopy of skeletal muscle (quadriceps) from Patient U12. Muscle architecture was normal with normal sarcomere structure **(A -I)**. Mitochondria were normally localised near the Z discs and most had normal structure. Some lipid droplets were noted but this was not deemed to be outside normal limits. Evidence of degraded mitochondria was noted, usually near the basal lamina, although this was likely artefactual **(E-F)**. A small proportion of mitochondria had a disorganised cristae structure, usually associated with close proximity to lipid droplets, of uncertain significance **(G-I)**. Scale bars: 2 µm **(A, B, F)**, 500 nm **(C, D, E, G, H, I)**.

Brain imaging: MRI demonstrated abnormal signal in the caudate and lentiform nuclei, abnormal signal in the midbrain and symmetrical scarring and signal abnormality involving the basal ganglia structures.

Treatment: Unknown.

Diagnosis and further investigations: Identified a novel homozygous missense variant (p.Leu356Arg) in exon 9 of *AFG3L2*, a component of the conserved m-AAA protease. This ATP-dependent proteolytic complex of the mitochondrial inner membrane degrades misfolded

proteins and regulates ribosome assembly. This variant is predicted to be deleterious and probably damaging by SIFT and PolyPhen-2, respectively. When comparing possible amino acids found at this position among the top 150 known homologues of AFG3L2, leucine is always found at this position thereby indicating its functional importance. Whilst autosomal recessive mutations in *AFG3L2* are known to cause spastic ataxia 5 (Pierson *et al.*, 2011). Patients typically present with early-onset ataxia with myoclonus and the loss of ability to ambulate independently. Some patients have been reported to develop lower extremity weakness with distal muscle atrophy and axonal sensorimotor neuropathy, oculomotor apraxia, dysarthria and dystonia. Similarly to patient U12, it has been reported that electron microscopy of skeletal muscle from patients with mutations in *AFG3L2* showed abnormalities with misplaced mitochondria associated with large lipid droplets (Pierson *et al.*, 2011).

Patient U5 (*SERAC1*)

Age and gender: 4 years, male.

Relevant family history: First child born to first cousin consanguineous Afghan parents.

Clinical presentation: Sat at 10 months, crawling at 12 months, able to stand and take a few steps at 18 months. Able to babble with double syllable babble although never developed any words. Following a febrile illness at 2 years he lost all skills and developed a progressive dyskinetic movement disorder with dystonia and choreoathetoid movements. Also affected by sensorineural deafness, renal tubular dysfunction and dysphagia with failure to thrive requiring a gastrostomy. Died at four years of age due to multi-organ failure associated with infection.

Biochemical/metabolic findings: Raised 3-methylglutaconate with normal 3-methylglutarate and intermittently mildly raised orotate. Strongly raised methionine and elevated proline, alanine, phenylalanine and tyrosine, possibly secondary to liver dysfunction. Occasionally mildly raised lactate. Normal mitochondrial respiratory chain enzymes and mitochondrial DNA sequencing.

Brain imaging: Progressive abnormal signal in the basal ganglia and cerebellum.

Treatment: Unknown.

Diagnosis and further investigations: One novel homozygous mutation was found in *SERAC1* (c.1850delinsCA) which is predicted to cause a frameshift and the subsequent introduction of a premature stop codon (p.Ile617ThrfsTer6). Mutations in this gene are known

to cause 3-methylglutaconic aciduria with deafness, encephalopathy, and Leigh-like features (MEGDEL) syndrome (Wortmann *et al.*, 2012).

Patient U6 (*PGAP2*)

Age and gender: 2 years, male.

Relevant family history: First child born to consanguineous parents from Kuwait.

Clinical presentation: Dysmorphic features including microcephaly, retro- and micrognathia and a cleft of the soft and hard palate. Organ malformations affected the brain (Dandy-Walker malformation), heart (doubly committed subarterial ventricular septal defect), lung (bilateral pulmonary hypoplasia) and gut (Hirschsprung's disease). Early-onset asymmetric tonic seizures which responded to levetiracetam, but were non-responsive to pyridoxine or pyridoxal 5'-phosphate. He also suffered central apnoeas, severe peripheral and bulbar neuropathy (motor more pronounced than sensory) and a very disordered sleep pattern. Initially some slow but definite developmental progress was observed, but by the end of the first year of life, he had static development and no speech with further regressions during intercurrent illnesses. Died due to intestinal failure following an acute enterocolitis.

Biochemical/metabolic findings: Persistent and marked hyperphosphatasia, hypoketotic hypoglycaemia and pan- hypogammaglobulinaemia. Normal transferrin isoelectric focussing.

Brain imaging: Dandy-Walker malformation.

Treatment: Levetiracetam and continual oxygen supplementation.

Diagnosis and further investigations: A novel homozygous missense mutation was identified in the fourth transmembrane domain of *PGAP2* (p.Ala187Val). Whilst this variant is reported at a minor allele frequency of 3/12052 (0.0024%), it is predicted to be deleterious and probably damaging by SIFT and PolyPhen-2, respectively. Sequence alignment of PGAP2 across species shows that Ala187 is conserved in mammals and birds but not zebrafish. The parents were both confirmed to be heterozygous for this mutation by Sanger sequencing. Mutations in this gene have been described recently to cause hyperphosphatasia with mental retardation syndrome III (Hansen *et al.*, 2013; Krawitz *et al.*, 2013). Many features described in these cases are also present in our patient including microcephaly, cleft palate, Dandy-Walker malformation, heart septal defect, Hirschsprung's disease, epilepsy, hypotonia, profound developmental delay, disordered sleep pattern and hyperphosphatasia.

Patient U7 (*ALDOB/ TPP1*)

Age and gender: 7 years, male.

Relevant family history: Brother sharing the same type-I transferrin isoelectric focussing pattern but neurodevelopmentally normal.

Clinical presentation: Sat at 9 months and independent walking at 23 months. Presenting features were speech and language delay, microcephaly, increased tone and hyper-reflexia in the lower limbs with equivocal plantar responses but no ankle clonus.

Biochemical/metabolic findings: Type-I transferrin isoelectric focussing pattern.

Brain imaging: Thin corpus callosum.

Treatment: Unknown.

Diagnosis and further investigations: A novel homozygous missense variant was identified in the *TPP1* gene (p.Gly296Asp) alongside a known pathogenic mutation in *ALDOB* (c.178C>T; p.Arg60Ter). The missense mutation in *TPP1* is predicted to be deleterious and probably damaging by SIFT and PolyPhen-2, respectively. Leukocyte tripeptidyl peptidase I activity was subsequently found to be at the upper boundary of the affected range (28 nmol/hr/mg protein; ref: 42 – 339).

Patient U8 (GALE)

Age and gender: 6 years, male.

Relevant family history: N/A

Clinical presentation: Neonatal jaundice and lethargy, deranged liver function tests and hypoglycaemic episodes which improved with supportive treatment. Bilateral sensorineural hearing loss. Within two hours of pneumococcal immunisation at 4 years, he became unwell with pyrexia and hypotonia, followed by recurrent upper and lower respiratory tract infections for several months.

Biochemical/metabolic findings: Abnormal liver function tests, hypoglycaemia. Low plasma IgA and IgM in the context of normal IgG, pneumococcus-specific antibodies and lymphocyte subsets, possibly reflective of transient hypogammaglobulinaemia of infancy. Type-I transferrin isoelectric focussing pattern.

Brain imaging: N/A

Treatment: Unknown.

Diagnosis and further investigations: Compound heterozygous mutations were found affecting adjacent amino acids in exon 3 of the *GALE* gene. The first (p.Val94Met) is a known pathogenic mutation (Wohlers *et al.*, 1999) and the second is a novel missense mutation (p.Gly95Asp). Both variants are predicted to be deleterious and probably damaging by SIFT and PolyPhen-2, respectively. When comparing possible amino acids found at this position among the top 150 known homologues of GALE, no alternative amino acids are found at either position indicating their functional importance. UDP-galactose 4’-epimerase activity was then measured in red blood cells and found to be undetectable.

REFERENCES

Hansen L, Tawamie H, Murakami Y, Mang Y, ur Rehman S, Buchert R, *et al.* Hypomorphic mutations in PGAP2, encoding a GPI-anchor-remodeling protein, cause autosomal-recessive intellectual disability. Am J Hum Genet 2013; 92(4): 575-83.

Krawitz PM, Murakami Y, Riess A, Hietala M, Kruger U, Zhu N, *et al.* PGAP2 mutations, affecting the GPI-anchor-synthesis pathway, cause hyperphosphatasia with mental retardation syndrome. Am J Hum Genet 2013; 92(4): 584-9.

Pierson TM, Adams D, Bonn F, Martinelli P, Cherukuri PF, Teer JK, *et al.* Whole-exome sequencing identifies homozygous AFG3L2 mutations in a spastic ataxia-neuropathy syndrome linked to mitochondrial m-AAA proteases. PLoS Genet 2011; 7(10): e1002325.

Wohlers TM, Christacos NC, Harreman MT, Fridovich-Keil JL. Identification and characterization of a mutation, in the human UDP-galactose-4-epimerase gene, associated with generalized epimerase-deficiency galactosemia. Am J Hum Genet 1999; 64(2): 462-70.

Wortmann SB, Vaz FM, Gardeitchik T, Vissers LE, Renkema GH, Schuurs-Hoeijmakers JH, *et al.* Mutations in the phospholipid remodeling gene SERAC1 impair mitochondrial function and intracellular cholesterol trafficking and cause dystonia and deafness. Nat Genet 2012; 44(7): 797-802.

Yoshida A, Kobayashi K, Manya H, Taniguchi K, Kano H, Mizuno M, *et al.* Muscular dystrophy and neuronal migration disorder caused by mutations in a glycosyltransferase, POMGnT1. Dev Cell 2001; 1(5): 717-24.
