# Supplementary material for: Advantages and pitfalls of an extended gene panel for investigating complex neurometabolic phenotypes
Source: Brain. 2016 Sep 6;139(11):2844–54. doi: 10.1093/brain/aww221 (PMC5091046; doi:10.1093/brain/aww221)
Supplement: Supplementary Data [file aww221_supplementary_data.zip › brain-2016-00692-File014.pdf]

Disease group / disease	Gene name
**1. Disorders of amino acid and peptide metabolism**	
**1.1. Urea cycle disorders and inherited hyperammonaemias**	
1.1.1. Carbamoylphosphate synthetase I deficiency	*CPS1*
1.1.2. N-Acetylglutamate synthetase deficiency	*NAGS*
1.1.3. Ornithine transcarbamylase deficiency	*OTC*
1.1.4. Citrullinaemia type1	*ASS1*
1.1.5. Argininosuccinic aciduria	*ASL*
1.1.6. Argininaemia	*ARG1*
1.1.7. HHH syndrome	*SLC25A15*
1.1.8. Citrullinemia Type 2	*SLC25A13*
1.1.9. Hyperinsulinemic hypoglycemia and hyperammonemia	*GLUD1*
1.1.10. Hyperammonemia	*CA5A*
**1.2. Organic acidurias**	
1.2.1. Glutaric aciduria	
1.2.1.1. Glutaric aciduria type I	*GCDH*
1.2.1.2. Glutaric aciduria type III	*C7orf10*
1.2.2. Propionic aciduria	*PCCA*<br>*PCCB*
1.2.3. Methylmalonic aciduria	
1.2.3.1. Methylmalonyl-CoA mutase deficiency	*MUT*
1.2.3.2. Methylmalonyl-CoA epimerase deficiency	*MCEE*
1.2.4. Isovaleric aciduria	*IVD*
1.2.5. Methylcrotonylglycinuria	*MCCC1*<br>*MCCC2*
1.2.6. Methylglutaconic aciduria	
1.2.6.1. Methylglutaconic aciduria type I	*AUH*
1.2.6.2. Methylglutaconic aciduria type II, Barth syndrome	*TAZ*
1.2.6.3. Methylglutaconic aciduria type III, Costeff syndrome	*OPA3*
1.2.6.4. Methylglutaconic aciduria type IV	
1.2.6.5. Methylglutaconic aciduria type V	*DNAJC19*
1.2.6.6. Methylglutaconic aciduria with deafness, encephalopathy and Leigh-like syndrome (MEGDEL)	*SERAC1*
1.2.7. 3-Hydroxy-3-methyl-glutaric aciduria	*HMGCL*
1.2.8. 2-Methylbutyric aciduria	*ACADSB*
1.2.9. 2-Methyl-3-hydroxybutyric aciduria, HSD10 disease	*HSD17B10*
1.2.10. 3-Oxothiolase deficiency	*ACAT1*
1.2.11. Isobutyric aciduria	*ACAD8*
1.2.12. Methacrylic aciduria	*HIBCH*
1.2.13. 3-Hydroxyisobutyric aciduria	*ALDH6A1*
1.2.14. Methylmalonate semialdehyde dehydrogenase deficiency	*ALDH6A1*
1.2.15. L-2-hydroxyglutaric aciduria	*L2HGDH*
1.2.16. D-2-hydroxyglutaric aciduria	
1.2.16.1. D-2-hydroxyglutarate dehydrogenase deficiency	*D2HGDH*
1.2.16.2. Mitochondrial isocitrate dehydrogenase deficiency	*IDH2*
1.2.17. Aminoacylase deficiency	
1.2.17.1. Aminoacylase 1 deficiency	*ACY1*
1.2.17.2. Aminoacylase 2 deficiency	*ASPA*

1.2.18.	Methylmalonate semialdehyde dehydrogenase deficiency	*ALDH6A1*
1.2.19.	Combined methylmalonic and malonic aciduria	*ACSF3*
1.2.20.	Malonyl-CoA decarboxylase deficiency	*MLYCD*

1.3. Disorders of the metabolism of branched-chain amino acids not classified as organic acidurias

1.3.1.	Branched-chain amino acid transferase	*BCAT1*<br>*BCAT2*
1.3.2.	Maple syrup urine disease	
1.3.2.1.	BCKD E1 alpha subunit of deficiency	*BCKDHA*
1.3.2.2.	BCKD E1 beta subunit of deficiency	*BCKDHB*
1.3.2.3.	Dihydrolipoamide branched chain transacylase deficiency	*DBT*

1.4. Disorders of phenylalanine or tyrosine metabolism

1.4.1.	Phenylalanine hydroxylase deficiency	*PAH*
1.4.2.	Tyrosinaemia type II	*TAT*
1.4.3.	4-hydroxyphenylpyruvate dioxygenase deficiency	*HPD*
S	Tyrosinaemia type III	
S	Hawkinsinuria	
1.4.4.	Alkaptonuria	*HGD*
1.4.5.	Tyrosinaemia type I	*FAH*

1.5. Disorders of the metabolism of sulphur amino acids

1.5.1.	Methionine adenosyltransferase I/III deficiency	*MAT1A*
1.5.2.	Glycine N-methyltransferase deficiency	*GNMT*
1.5.3.	S-adenosylhomocysteine hydrolase deficiency	*AHCY*
1.5.4.	Cystathionine beta-synthase deficiency	*CBS*
1.5.5.	Cystathionase deficiency	*CTH*
1.5.6.	Isolated sulfite oxidase deficiency	*SUOX*
1.5.7.	Methionine synthase deficiency-cblG	*MTR*
1.5.8.	Methionine synthase reductase deficiency-cblE	*MTRR*

1.6. Disorders of histidine, tryptophan or lysine metabolism

1.6.1.	Histidinaemia	*HAL*
1.6.2.	Urocanase deficiency	*UROC1*
1.6.3.	Glutamate formiminotransferase deficiency	*FTCD*
1.6.4.	Tryptophanaemia	*TDO2*
1.6.5.	Hyperlysinaemia	*AASS*<br>*PTPRZ1*
1.6.6.	2-Aminoadipic aciduria	*DHTKD1*
1.6.7.	2-Oxoadipic aciduria	*DHTKD1*
1.6.8.	Hydroxykynureninuria	*KYNU*
1.6.9.	Hydroxylysinuria	*AGPHD1*<br>*AGXT2L2*

1.7. Disorders of serine, glycine or glycerate metabolism

1.7.1.	Phosphoglycerate dehydrogenase deficiency	*PHGDH*
1.7.2.	Phosphoserine phosphatase deficiency	*PSPH*
1.7.3.	Phosphoserine aminotransferase deficiency	*PSAT1*
1.7.4.	Nonketotic hyperglycinaemia	
1.7.4.1.	P protein deficiency	*GLDC*
1.7.4.2.	T protein deficiency	*AMT*
1.7.4.3.	H protein deficiency	*GCSH*
1.7.5.	Sarcosinaemia	*SARDH*

1.7.6.	D-glyceric aciduria	*GLYCTK*

1.8. Disorders of ornithine or proline metabolism

1.8.1.	Ornithine aminotransferase deficiency	*OAT*
1.8.2.	Hyperprolinaemia type I	*PRODH*
1.8.3.	Hyperprolinaemia type II	*ALDH4A1*
1.8.4.	Hypoprolinaemia, Cutis laxa, autosomal recessive, type IIIa	*ALDH18A1*
1.8.5.	Cutis laxa, autosomal recessive, type IIb/IIIb	*PYCR1*

1.9. Disorders of amino acid transport

1.9.1.	Lysinuric protein intolerance	*SLC7A7*
1.9.2.	Cystinuria	*SLC3A1*<br>*SLC7A9*
1.9.3.	Cystinuria-hypotonia syndrome (contiguous gene defect)	
1.9.4.	Hartnup disease	*SLC6A19*
1.9.5.	Iminoglycinuria	*SLC36A2*
1.9.6.	Lowe syndrome	*OCRL*
1.9.7.	Hypotonia-cystinuria syndrome	*SLC3A1*
1.9.8.	Hypotonia-cystinuria syndrome	*PREPL*

1.10. Other disorders of amino acid metabolism

1.10.1.	Glutamine deficiency, congenital	*GLUL*

1.11. Disorders of the gamma-glutamyl cycle

1.11.1.	Glutathionuria	*GGT1*
1.11.2.	Cysteinylglycinase deficiency	*DPEP1*
1.11.3.	Oxoprolinuria	*OPLAH*
1.11.4.	Gamma-glutamylcysteine synthetase deficiency	*GCLC*
1.11.5.	Glutathione synthetase deficiency	*GSS*

1.12. Other disorders of peptide metabolism

1.12.1.	Prolidase deficiency	*PEPD*
1.12.2.	Carnosinaemia	*CNDP1*
1.12.3.	Homocarnosinosis	

1.13. Other disorders of amino acid and protein metabolism

2. Disorders of carbohydrate metabolism

2.1. Disorders of galactose metabolism

2.1.1.	Classical galactosaemia	*GALT*
2.1.2.	Galactokinase deficiency	*GALK1*
2.1.3.	Uridine diphosphate galactose-4-epimerase deficiency	*GALE*

2.2. Disorders of fructose metabolism

2.2.1.	Essential fructosuria	*KHK*
2.2.2.	Hereditary fructose intolerance	*ALDOB*

2.3. Disorders of pentose metabolism

2.3.1.	Essential pentosuria	*DCXR*
2.3.2.	Ribose-5-phosphate isomerase deficiency	*RPIA*
2.3.3.	Transaldolase deficiency	*TALDO1*

2.4. Disorders of glycerol metabolism

2.4.1.	Glycerol kinase deficiency	*GK*
2.4.2.	Complex glycerol kinase deficiency due to contiguous gene deletion	

2.5. Disorders of glyoxylate metabolism

2.5.1. Primary hyperoxaluria type I — *AGXT*
2.5.2. Primary hyperoxaluria type II — *GRHPR*

2.6. Disorders of glucose transport

2.6.1. Glucose transporter 1 deficiency (blood-brain barrier) — *SLC2A1*
2.6.2. Glucose transporter 2 deficiency — *SLC2A2*
S Fanconi-Bickel syndrome
2.6.3. Glucose/galactose malabsorption — *SLC5A1*

2.7. Disorders of gluconeogenesis

2.7.1. Fructose-1,6-bisphosphatase deficiency — *FBP1*
2.7.2. Pyruvate carboxylase deficiency — *PC*
2.7.3. Phosphoenolpyruvate carboxykinase deficiency — *PCK1*

2.8. Glycogen storage disorders

2.8.1. Glycogen storage disease type 1a, von Gierke — *G6PC*
2.8.2. Glycogen storage disease type 1b, von Gierke — *SLC37A4*
2.8.3. Glycogen storage disease type II, Pompe — *GAA*
2.8.4. Glycogen storage disease type III, Cori — *AGL*
2.8.5. Glycogen storage disease type IV, Andersen — *GBE1*
2.8.6. Glycogen storage disease type V, McArdle — *PYGM*
2.8.7. Glycogen storage disease type VI, Hers — *PYGL*
2.8.8. Glycogen storage disease type VII, Tarui — *PFKM*
2.8.9. Glycogen storage disease type IX
2.8.9.1. Hepatic phosphorylase kinase deficiency — *PHKA2*
2.8.9.2. Hepatic and muscle phosphorylase kinase deficiency — *PHKB*
2.8.9.3. Hepatic phosphorylase kinase deficiency with cirrhosis — *PHKG2*
2.8.9.4. Muscle phosphorylase kinase deficiency — *PHKA1*
2.8.9.5. Cardiac muscle phosphorylase kinase deficiency — *PRKAG2*
2.8.10. Glycogen storage disease type X — *PGAM2*
2.8.11. Glycogen storage disease type XI — *SLC2A2*
2.8.12. Glycogen storage disease type XIV — *PGM1*
2.8.13. Glycogen storage disease type XV — *GYG1*
2.8.14. Glycogen storage disease type 0a, liver — *GYS2*
2.8.15. Glycogen storage disease type 0b, muscle — *GYS1*
2.8.16. Other glycogen storage disease
2.8.16.1. Muscle LDH deficiency — *LDHA*
2.8.16.2. Aldolase A deficiency — *ALDOA*
2.8.16.3. Beta-enolase deficiency — *ENO3*
2.8.16.4. Phosphoglycerate kinase deficiency — *PGK1*
2.8.17. Unspecified glycogen storage disease

2.9. Other carbohydrate disorders

2.9.1. Lactose intolerance — *LCT*
2.9.2. Disaccharide intolerance 1 — *SI*
2.9.3. Trehalase deficiency — *TREH*

3. Disorders of fatty acid and ketone body metabolism

3.1. Disorders of lipolysis

3.1.1. Neutral lipid storage disease — *ABHD5*

3.2. Disorders of carnitine transport and the carnitine cycle

3.2.1. Carnitine transporter deficiency — *SLC22A5*
3.2.2. Carnitine palmitoyltransferase I (CPTI) deficiency — *CPT1A*

3.2.3.	Carnitine acylcarnitine translocase deficiency	*SLC25A20*
3.2.4.	Carnitine palmitoyltransferase II (CPTII) deficiency	*CPT2*

3.3. Disorders of mitochondrial fatty acid oxidation

3.3.1.	Very long - chain acyl CoA dehydrogenase deficiency	*ACADVL*
3.3.2.	Mitochondrial trifunctional protein deficiency	*HADHA*<br>*HADHB*
3.3.3.	Medium - chain acyl CoA dehydrogenase deficiency	*ACADM*
3.3.4.	Short - chain acyl CoA dehydrogenase deficiency	*ACADS*
3.3.5.	3-alpha-hydroxyacyl- CoA dehydrogenase deficiency	*HADH*
3.3.6.	Multiple acyl-CoA dehydrogenase deficiency	
3.3.6.1.	Electron transfer flavoprotein deficiency, alpha chain	*ETFA*
3.3.6.2.	Electron transfer flavoprotein deficiency, beta chain	*ETFB*
3.3.6.3.	ETF-ubiquinone oxidoreductase deficiency	*ETFDH*

3.4. Disorders of ketone body metabolism

3.4.1.	3-Hydroxy-3-Methylglutaryl-CoA synthase deficieny	*HMGCS2*
3.4.2.	Succinyl-CoA:3-Oxoacid-CoA transferase (SCOT) deficiency	*OXCT1*
3.4.3.	Cytosolic acetoacetyl-CoA thiolase deficiency	*ACAT1*

3.5. Other disorders of fatty acid and ketone body metabolism

3.5.1.	Malonyl CoA decarboxylase deficiency	*MLYCD*

4. Disorders of energy metabolism

4.1. Disorders of pyruvate metabolism

4.1.1.	Pyruvate dehydrogenase complex deficiency	
4.1.1.1.	Pyruvate dehydrogenase E1α subunit deficiency	*PDHA1*
4.1.1.2.	Pyruvate dehydrogenase E1β subunit deficiency	*PDHB*
4.1.1.3.	Dihydrolipoyl transacetylase deficiency	*DLAT*
4.1.1.4.	Dihydrolipoyl dehydrogenase deficiency	*DLD*
4.1.1.5.	Pyruvate dehydrogenase E3 binding protein deficiency	*PDHX*
4.1.1.6.	Pyruvate dehydrogenase kinase deficiency	*PDK1*<br>*PDK2*<br>*PDK3*<br>*PDK4*
4.1.1.7.	Pyruvate dehydrogenase phosphatase deficiency	*PDP1*<br>*PDP2*<br>*PDPR*
4.1.1.8.	Pyruvate dehydrogenase deficiency, unspecified	

4.2. Disorders of the citric acid cycle

4.2.1.	2-Oxoglutarate dehydrogenase deficiency	*OGDH*<br>*DLST*
4.2.2.	Fumarase deficiency	*FH*

4.3. Mitochondrial respiratory chain disorders (caused by nuclear mutations only)

4.3.1.	OXPHOS structural subunits	
4.3.1.1.	Complex I	*NDUFS1*<br>*NDUFS2*<br>*NDUFS3*<br>*NDUFS4*<br>*NDUFS6*<br>*NDUFS7*<br>*NDUFS8*

		NDUFV1
		NDUFV2
		NDUFA1
		NDUFA2
		NDUFA9
		NDUFA10
		NDUFA11
		NDUFA12
		NDUFB3
		NDUFB9
4.3.1.2.	Complex II	*SDHA*
		SDHB
		SDHC
		SDHD
4.3.1.3.	Complex III	*UQCRB*
		UQCRQ
4.3.1.4.	Complex IV	*COX4I2*
		COX6B1
		COX7B
4.3.1.4.	Complex V	*ATP5E*
		ATP5A1
4.3.2.	OXPHOS assembly factors	
4.3.2.1.	Complex I	*NDUFAF1*
		NDUFAF2
		NDUFAF3
		NDUFAF4
		NDUFAF6
		NDUFAF5
		NUBPL
		FOXRED1
		ACAD9
4.3.2.2.	Complex II	*SDHAF1*
		SDHAF2
4.3.2.3.	Complex III	*BCS1L*
		HCCS
		TTC19
4.3.2.4.	Complex IV	*SURF1*
		SCO2
		SCO1
		COX10
		COX15
		LRPPRC
		FASTKD2
		ETHE1
		TACO1
		COA5
		COX14
		COX20
4.3.2.5.	Complex V	*ATPAF2*

		TMEM70
4.3.3.	Required for mtDNA maintenance	*POLG*
		POLG2
		C10orf2
		SLC25A4
		TYMP
		DGUOK
		TK2
		SUCLA2
		SUCLG1
		MPV17
		RRM2B
4.3.4.	Required for mitochondrial gene expression	*PUS1*
		MTO1
		MRPS16
		MRPS22
		MRPL3
		GFM1
		TSFM
		TUFM
		AARS2
		DARS2
		EARS2
		FARS2
		HARS2
		IARS2
		LARS2
		MARS2
		RARS2
		SARS2
		YARS2
		TRMU
		MTFMT
		MTPAP
		C12orf65
		RMND1
4.3.5.	Defective Fe-S/lipoic acid biosynthesis	*ISCU*
		FXN
		NFU1
		BOLA3
		LIAS
		ABCB7
		GLRX5
4.3.6.	Disorders of CoQ10 biosynthesis	*PDSS1*
		PDSS2
		COQ2
		ADCK3
		COQ9
		COQ6

		COQ4
4.3.7.	Secondary CoQ10 deficiency	*APTX*
		SETX
		ETFDH
4.3.8.	Disorders of mitochondrial solute import	*SLC25A3*
		SLC25A12
		SLC25A22
		SLC25A38
4.3.9.	Disorders of mitochondrial protein import	*TIMM8A*
		DNAJC19
		GFER
		PNPT1
4.3.10.	Disorders of mitochondrial membrane lipids	*TAZ*
		AGK
		SERAC1
4.3.11.	Disorders of mitochondrial dynamics, fusion and fission	*MFN2*
		OPA1
		DNM1L
		MFF
4.3.12.	Miscellaneous disorders/unknown function	*AIFM1*
		TMEM126A
		SPG7
		HSPD1
		AFG3L2
4.3.12.1.	Hyperoxaluria Type III	*HOGA1*
4.3.12.1.	Charcot-Marie-Tooth disease, recessive intermediate, B (Lysyl-tRNA synthetase mutations)	*KARS*
4.3.12.1.	Spinocerebellar ataxia-7	*ATXN7*
4.3.12.1.	Succinyl CoA:3-oxoacid CoA transferase deficiency	*OXCT1*
4.3.12.1.	Parkinson disease 6, early onset	*PINK1*
4.3.12.1.	Hypotonia-cystinuria syndrome	*PPM1B*
4.3.12.1.	Wolfram syndrome 1	*WFS1*
4.3.12.1.	Wolfram syndrome 2	*CISD2*
4.3.13.	Disorders of creatinine metabolism	
4.3.13.1.	Creatine transporter deficiency	*SLC6A8*
4.3.13.2.	Guanidinoacetate methyltransferase deficiency	*GAMT*
4.3.13.3.	Arginine:glycine amidinotransferase deficiency	*GATM*

5. Disorders in the metabolism of purines, pyrimidines and nucleotides

5.1. Disorders of purine metabolism

5.1.1.	Primary idiopathic gout	*ABCG2*
5.1.2.	Familial juvenile hyperuricaemic nephropathy	*UMOD*
5.1.3.	Adenylosuccinate lyase deficiency	*ADSL*
5.1.4.	AICAR transformylase deficiency	*ATIC*
5.1.5.	Adenosine deaminase deficiency	*ADA*
5.1.6.	Deoxyguanosine kinase deficiency	*DGUOK*
5.1.7.	Myoadenylate deaminase deficiency	*AMPD1*
5.1.8.	Lesch-Nyhan syndrome	*HPRT1*
5.1.9.	Adenine phosphoribosyl transferase deficiency	*APRT*

5.1.10.	Phosphoribosyl pyrophosphate synthetase 1 defects	*PRPS1*
5.1.11.	Inosine triphosphatase deficiency	*ITPA*
5.1.12.	Adenosine deaminase superactivity	
5.1.13.	Purine nucleoside phosphorylase deficiency	*PNP*
5.1.14.	Mitochondrial Ribonucelotide Reductase subunit 2 deficiency	*RRM2B*
5.1.15.	Xanthinuria type I	*XDH*
5.1.16.	Xanthinuria type II	*XDH*
		AOX1
5.1.17.	Thiopurine S-methyltransferase deficiency	*TPMT*

5.2. Disorders of pyrimidine metabolism

5.2.1.	Orotic aciduria	*UMPS*
5.2.2.	Pyrimidine - 5 - nucleotidase deficiency	*NT5C*
5.2.3.	Dihydroorotate dehydrogenase deficiency	*DHODH*
5.2.4.	Uridine-5'-monophosphate hydrolase superactivity	*NT5C3*
5.2.5.	Thymidine phosphorylase deficiency	*TYMP*
5.2.6.	Thymidine kinase 2 deficiency	*TK2*
5.2.7.	Dihydropyrimidine dehydrogenase deficiency	*DPYD*
5.2.8.	Dihydropyrimidinase deficiency	*DPYS*
5.2.9.	Beta-ureidopropionase deficiency	*UPB1*
5.2.10.	Hyper-beta-alaninaemia	
5.2.11.	Beta-aminoisobutyrate-pyruvate transaminase deficiency	

5.3. Disorders of nucleotide metabolism

5.3.1.	Aicardi-Goutières Syndrome (AGS)	
5.3.1.1.	AGS1	*TREX1*
5.3.1.2.	AGS2	*RNASEH2B*
5.3.1.3.	AGS3	*RNASEH2C*
5.3.1.4.	AGS4	*RNASEH2A*
5.3.1.5.	AGS5	*SAMHD1*
5.3.1.6.	AGS6	*ADAR*
5.3.2.	RNASET2-deficient cystic leukoencephalopathy	*RNASET2*

6. Disorders of the metabolism of sterols

6.1. Disorders of sterol biosynthesis

6.1.1.	Mevalonate kinase deficiency	*MVK*
6.1.2.	Smith - Lemli - Opitz syndrome	*DHCR7*
6.1.3.	X-linked dominant chondrodysplasia punctata 2	*EBP*
6.1.4.	Congenital hemidysplasia with ichtyosiform erythroderma and limb defects	*NSDHL*
6.1.5.	Desmosterolosis	*DHCR24*
6.1.6.	Lathosterolosis	*SC5DL*
6.1.7.	Greenberg skeletal dysplasia	*LBR*
6.1.8.	Antley-Bixler syndrome	
6.1.8.1.	Antley-Bixler syndrome with disordered steroidogenesis	*POR*
6.1.8.2.	Antley-Bixler syndrome type without disordered steroidogenesis	*FGFR2*
6.1.9.	Sterol-C4-methyl oxidase deficiency	*MSMO1*

6.2. Disorders of bile acid biosynthesis

6.2.1.	3- β-hydroxysterol Δ5-oxidoreductase/isomerase deficiency	*HSD3B7*
6.2.2.	Δ4-3-oxysterol 5β-reductase deficiency	*AKR1D1*
6.2.3.	Oxysterol 7-alpha-hydroxylase deficiency	*CYP7B1*
6.2.4.	Cholesterol 7-alpha-hydroxylase deficiency	*CYP7A1*

6.2.5.	Cerebrotendinous xanthomatosis	*CYP27A1*
6.2.6.	Bile acid amidation defect	*BAAT*
6.2.7.	Bile acid CoA ligase deficiency	*SLC27A5*

6.3. Disorders of bile acid metabolism and transport

6.3.1.	Bilirubin UDP-glucuronosyltransferase 1 deficiency	*UGT1A1*
6.3.2.	Byler disease	*ATP8B1*
6.3.3.	Progressive familial intrahepatic cholestasis type 2	*ABCB11*
6.3.4.	Progressive familial intrahepatic cholestasis type 3	*ABCB4*

6.4. Other disorders in the metabolism of sterols

6.4.1.	X-linked ichthyosis	*STS*

7. Disorders of porphyrin and haem metabolism

7.1.1.	Acute neuropathic porphyrias	
7.1.1.1.	Acute intermittent porphyria	*HMBS*
7.1.1.2.	Variegate porphyria	*PPOX*
7.1.1.3.	Hereditary coproporphyria	*CPOX*
7.1.1.4.	Acute hepatic porphyria	*ALAD*
7.1.2.	Porphyrias with erosive photodermatosis	
7.1.2.1.	Porphyria cutanea tarda	*UROD*
7.1.2.2.	Congenital erythropoietic porphyria	*UROS*
7.1.3.	Porphyrias with acute painful photosensitivity	
7.1.3.1.	Erythropoietic protoporphyria	*FECH*
7.1.3.2.	X-linked dominant protoporphyria	*ALAS2*
7.1.3.3.	X-linked sideroblastic anaemia (XLSA)	*ALAS2*

8. Disorders of lipid and lipoprotein metabolism

8.1. Inherited hypercholesterolaemias

8.1.1.	Disorder of low density lipoprotein receptor	*LDLR*
8.1.2.	Sitosterolaemia	*ABCG5*<br>*ABCG8*
8.2.1.	Autosomal dominant hypercholesterolemia-3	*PCSK9*
8.2.1.	Autosomal recessive hypercholesterolemia	*LDLRAP1*

8.2. Inherited hypertriglyceridaemias

8.2.1.	Familial chylomicronaemia	
8.2.1.1.	Familial lipoprotein lipase deficiency	*LPL*
8.2.1.2.	Familial apolipoprotein C - II deficiency	*APOC2*
8.2.2.	Familial hypertriglyceridaemia	*APOA5*<br>*LIPI*

8.3. Inherited mixed hyperlipidaemias

8.3.1.	Familial dysbetalipoproteinaemia	*APOE*
8.3.2.	Familial combined hyperlipoproteinaemia	*USF1*
8.3.3.	Hepatic lipase deficiency	*LIPC*

8.4. Disorders of high density lipoprotein metabolism

8.4.1.	Apolipoprotein A-I deficiency	*APOA1*
8.4.2.	Tangier disease	*ABCA1*
8.4.3.	Lecithin cholesterol acyltransferase deficiency	*LCAT*
8.4.3.1.	Fish-eye disease	
8.4.3.2.	Norum disease	
8.4.4.	Familial hyperalphalipoproteinaemia	*CETP*

8.5. Inherited hypolipidaemias

8.5.1.	Familial abetalipoproteinaemia	*MTTP*
8.5.2.	Familial hypobetalipoproteinaemia	*APOB*
8.5.3.	Anderson disease	*SAR1B*
8.5.3.	Scavenger receptor class B type I deficiency	*SCARB1*

8.6. Other disorders of lipid and lipoprotein metabolism

8.6.1.1.	Sjøgren - Larsson syndrome	*ALDH3A2*
8.6.1.2.	Pancreatic triacylglycerol lipase deficiency	*PNLIP*
8.6.1.3.	Pancreatic colipase deficiency	*CLPS*

8.7. Unspecified disorders of lipid and lipoprotein metabolism

8.8. Disorders of complex lipid synthesis

8.8.1.	Serine palmitoyl transferase deficiency	*SPTLC1*
8.8.2.	Serine palmitoyl transferase deficiency	*SPTLC2*
8.8.3.	Fatty acid 2-hydroxylase deficiency	*FA2H*
8.8.4.	Phosphatidate phosphatase deficiency	*LPIN1*
8.8.5.	Phospholipase A2 deficiency	*PLA2G6*
8.8.6.	PHARC syndrome	*ABHD12*
8.8.7.	Choline kinase deficiency	*CHKB*
8.8.8.	GM3 synthase deficiency	*ST3GAL5*
8.8.9.	Acylglycerol kinase deficiency (Senger syndrome)	*AGK*

9. Congenital disorders of glycosylation and other disorders of protein modification

S CDG

9.1. Disorders of protein N-glycosylation

9.1.1.	Phosphomannomutase 2 deficiency	*PMM2*
9.1.2.	Phosphomannose isomerase deficiency	*MPI*
9.1.3.	Glucosyltransferase 1 deficiency	*ALG6*
9.1.4.	Mannosyltransferase 6 deficiency	*ALG3*
9.1.5.	Mannosyltransferase 8 deficiency	*ALG12*
9.1.6.	Glucosyltransferase 2 deficiency	*ALG8*
9.1.7.	Mannosyltransferase 2 deficiency	*ALG2*
9.1.8.	UDP-GlcNAc:Dol-P-GlcNac-P transferase deficiency	*DPAGT1*
9.1.9.	Mannosyltransferase 1 deficiency	*ALG1*
9.1.10.	Mannosyltransferase 7-9 deficiency	*ALG9*
9.1.11.	Flippase of Man5GlcNAc2-PP-Dol deficiency	*RFT1*
9.1.12.	N-acetylglucosaminyltransferase deficiency	*MGAT2*
9.1.13.	Glucosidase 1 deficiency	*GLS*
9.1.14.	TUSC3-CDG	*TUSC3*
9.1.15.	SRD5A3-CDG	*SRD5A3*
9.1.16.	Mannosyltransferase 1 deficiency	*ALG1*
9.1.17.	Congenital myasthenic sydrome	*ALG14*
9.1.17.	Congenital myasthenic sydrome	*GFPT1*
9.1.17.	ALG13-CDG	*ALG13*
9.1.17.	ALG11-CDG	*ALG11*
9.1.17.	ALG3-CDG	*ALG3*
9.1.17.	ALG9-CDG	*ALG9*
9.1.17.	IAP-CDG	*MAGT1*
9.1.17.	MOGS-CDG	*MOGS*
9.1.17.	MAN1B1-CDG	*MAN1B1*

9.1.17. ST3GAL3-CDG *ST3GAL3*

9.2. Disorders of protein O-glycosylation

9.2.1. O-xylosylglycan synthesis deficiencies

9.2.1.1. Multiple exostoses type I *EXT1*
9.2.1.2. Multiple exostoses type II *EXT2*
9.2.1.3. Beta-1,4-galactosyltransferase 7 deficiency *B4GALT7*

9.2.2. O-N-acetylgalactosaminylglycan synthesis deficiencies

9.2.2.1. Polypeptide N-acetylgalactosaminyl transferase deficiency *GALNT3*
9.2.2.2. GALNT12-CDG *GALNT12*
9.2.2.3. COSMC-CDG *C1GALT1C1*

9.2.3. O-xylosyl/N-acetylgalactosaminylglycan synthesis deficiencies *SLC35D1*

9.2.4. O-mannosylglycan synthesis deficiencies

9.2.4.1. Protein-O-mannosyltransferase 1 deficiency *POMT1*
9.2.4.2. Protein-O-mannosyltransferase 2 deficiency *POMT2*
9.2.4.3. Protein-O-mannose beta-1,2-N-acetyglucosaminyltransferase deficiency *POMGNT1*
9.2.4.4. Fukutin deficiency *FKTN*
9.2.4.5. Fukutin-related protein deficiency *FKRP*
9.2.4.6. N-acetylglucosaminyltransferase-like protein deficiency *LARGE*
9.2.4.7. O-fucose-specific beta-1,3-N-acetylglucosaminyltransferase deficiency *LFNG*
9.2.4.8. O-fucose-specific beta-1,3-N-glucosyltransferase deficiency *B3GALTL*
9.2.4.8. LFNG-CDG *LFNG*
9.2.4.8. B4GALT7-CDG *B4GALT7*
9.2.4.8. B3GAT3-CDG *B3GAT3*
9.2.4.8. CHSY1-CDG *CHSY1*
9.2.4.8. CHST3-CDG *CHST3*
9.2.4.8. CHST14-CDG *CHST14*
9.2.4.8. CHST6-CDG *CHST6*

9.3. Disorders of glycosphingolipid and glycosylphosphatidylinositol anchor glycosylation

9.3.1.1. Lactosylceramide alpha-2,3-sialyltransferase deficiency *ST3GAL5*
9.3.1.2. Phosphatidylinositolglycan, class M deficiency *PIGM*
9.3.1.3. Hyperphosphatasia *PIGV*
9.3.1.4. Hyperphosphatasia *PIGO*
9.3.1.4. PIGA-CDG *PIGA*
9.3.1.4. PIGL-CDG *PIGL*
9.3.1.4. PIGN-CDG *PIGN*
9.3.1.4. PGAP2-CDG *PGAP2*

9.4. Disorders of multiple glycosylation and other glycosylation pathways

9.4.1. GDP-Man:Dol-P mannosyltransferase deficiency *DPM1*
9.4.2. Lec35 deficiency *MPDU1*
9.4.3. Beta-1,4-galactosyltransferase 1 deficiency *B4GALT1*
9.4.4. UDP-GlcNAc epimerase/kinase deficiency *GNE*
9.4.5. CMP-sialic acid transporter deficiency *SLC35A1*
9.4.6. GDP-fucose transporter deficiency *SLC35C1*
9.4.7. Dolichol pathway deficiencies

9.4.7.1. Dolichol kinase deficiency *DOLK*

9.4.8. Conserved oligomeric Golgi (COG) complex deficiency

9.4.8.1. Component of COG complex 7 deficiency *COG7*
9.4.8.2. Component of COG complex 1 deficiency *COG1*

9.4.8.3.	Component of COG complex 8 deficiency	*COG8*
9.4.8.3.	Component of COG complex 4 deficiency	*COG4*
9.4.8.3.	Component of COG complex 5 deficiency	*COG5*
9.4.8.3.	Component of COG complex 6 deficiency	*COG6*
9.4.9.	V-ATPase deficiencies	
9.4.9.1.	V0 subunit A2 of vesicular H(+)-ATPase deficiency	*ATP6V0A2*
9.4.9.2.	COPII component SEC23B	*SEC23B*

9.5. Disorders of protein ubiquitinylation

9.6. Other disorders of protein modification

9.6. Other CDGs

9.6.1.	SLC35A2-CDG	*SLC35A2*
9.6.1.	G6PC3-CDG	*G6PC3*
9.6.1.	CDG2K	*TMEM165*
9.6.1.	Retinitis pigmentosa	*DHDDS*
9.6.1.	DMP3-CDG	*DPM3*

10. Lysosomal disorders

10.1. Mucopolysaccharidoses

10.1.1.	MPS I, Hurler, Scheie disease	*IDUA*
10.1.2.	MPS II, Hunter disease	*IDS*
10.1.3.	MPS III, Sanfilippo disease	
10.1.3.1.	MPS IIIA, Sanfilippo A disease	*SGSH*
10.1.3.2.	MPS IIIB, Sanfilippo B disease	*NAGLU*
10.1.3.3.	MPS IIIC, Sanfilippo C disease	*HGSNAT*
10.1.3.4.	MPS IIID, Sanfilippo D disease	*GNS*
10.1.4.	MPS IV, Morquio disease	
10.1.4.1.	MPS IVA, Morquio A disease	*GALNS*
10.1.4.2.	MPS IVB, Morquio B disease	*GLB1*
10.1.5.	MPS VI, Maroteaux - Lamy disease	*ARSB*
10.1.6.	MPS VII, Sly disease	*GUSB*
10.1.7.	MPS IX, Natowicz	*HYAL1*

10.2. Oligosaccharidoses

10.2.1.	Alpha - D – mannosidosis	*MAN2B1*
10.2.2.	Beta - D – mannosidosis	*MANBA*
10.2.3.	Sialidosis	*NEU1*
10.2.4.	Aspartylglucosaminuria	*AGA*
10.2.5.	Fucosidosis	*FUCA1*
10.2.6.	Schindler disease	*NAGA*

10.3. Sphingolipidoses

10.3.1.	GM1-gangliosidosis	*GLB1*
10.3.2.	GM2-gangliosidosis	
10.3.2.1.	GM2-gangliosidosis 0-variant, Sandhoff disease	*HEXB*
10.3.2.2.	GM2-gangliosidosis B-variant, Tay-Sachs disease	*HEXA*
10.3.2.3.	GM2-gangliosidosis AB-variant	*GM2A*
10.3.3.	Gaucher disease	*GBA*
10.3.4.	Krabbe disease	*GALC*
10.3.5.	Metachromatic leukodystrophy	*ARSA*
10.3.6.	Prosaposin deficiency	*PSAP*
10.3.6.1.	Saposin A deficiency	

10.3.6.2. Saposin B deficiency	
10.3.6.3. Saposin C deficiency	
10.3.6.4. Saposin D deficiency	
10.3.7. Fabry disease	*GLA*
10.3.8. Farber disease	*ASAH1*
10.3.9. Niemann-Pick disease type A or B	*SMPD1*
10.3.10. Niemann-Pick disease type C	
10.3.10.1. Niemann-Pick disease type C1	*NPC1*
10.3.10.2. Niemann-Pick disease type C2	*NPC2*

10.4. Ceroid lipfuscinoses, neuronal (CLN)

10.4.1. CLN1, Santavuori-Haltia disease	*PPT1*
10.4.2. CLN2, Jansky-Bielschowsky disease	*TPP1*
10.4.3. CLN3, Batten Spielmeyer-Vogt disease	*CLN3*
10.4.4. CLN4A, Kufs disease recessive type	*CLN6*
10.4.5. CLN4B Kufs disease dominant type	*DNAJC5*
10.4.6. CLN5 Finnish variant	*CLN5*
10.4.7. CLN6	*CLN6*
10.4.8. CLN7	*MFSD8*
10.4.9. CLN8, Northern epilepsy type	*CLN8*
10.4.10. CLN9	
10.4.11. CLN10	*CTSD*

10.5. Lysosomal export disorders

10.5.1. Cystinosis	*CTNS*
10.5.2. Salla disease/infantile sialic acid storage disease	*SLC17A5*

10.6. Other lysosomal disorders

10.6.1. Mucolipidosis II, I-cell disease	*GNPTAB*
10.6.2. Mucolipidosis III, Pseudo-Hurler polydystrophy	*GNPTG*
10.6.3. Mucolipidosis IV	*MCOLN1*
10.6.4. Multiple sulphatase deficiency	*SUMF1*
10.6.5. Wolman/cholesterol ester storage disease	*LIPA*
10.6.6. Pompe disease, GSD type II	*GAA*
10.6.7. Sialuria	*GNE*
10.6.8. Danon disease	*LAMP2*
10.6.9. Cathepsin-related disorders	
10.6.9.1. Galactosialidosis	*CTSA*
10.6.9.2. Papillon-Lefèvre syndrome	*CTSC*
10.6.9.3. Pycnodysostosis	*CTSK*
10.6.10. Hermansky-Pudlak Syndrome	*HPS1*

11. Peroxisomal disorders

11.1. Disorders of peroxisome biogenesis

PEX1
PEX2
PEX3
PEX5
PEX6
PEX10
PEX12
PEX13
PEX14

	PEX16
	PEX19
	PEX26

11.2. Rhizomelic chondrodysplasia punctata

11.2.1. Rhizomelic chondrodysplasia punctata type 1	*PEX7*
11.2.2. Rhizomelic chondrodysplasia punctata type 2	*GNPAT*
11.2.3. Rhizomelic chondrodysplasia punctata type 3	*AGPS*

11.3. Disorders of peroxisomal alpha-, beta and omega-oxidation

11.3.1. X-linked adrenoleukodystrophy	*ABCD1*
11.3.2. Peroxisomal acyl-CoA oxidase 1 deficiency	*ACOX1*
11.3.3. Peroxisomal D-bifunctional protein deficiency	*HSD17B4*
11.3.4. Sterol carrier protein deficiency	*SCP2*
11.3.5. Alpha-methylacyl-CoA racemase deficiency	*AMACR*
11.3.6. Refsum disease	*PHYH*

11.4. Other peroxisomal disorders

11.4.1. Primary hyperoxaluria type I	*AGXT*
11.4.2. Acatalasaemia	*CAT*
11.4.3. Mulibrey nanism	*TRIM37*

12. Disorders of neurotransmitter metabolism

12.1. Disorders in the metabolism of biogenic amines

12.1.1. Tyrosine hydroxylase deficiency	*TH*
12.1.2. Aromatic L-amino acid decarboxylase deficiency	*DDC*
12.1.3. Dopamine beta-hydroxylase deficiency	*DBH*
12.1.4. Monoamine oxidase	*MAOA*

12.2. Disorders in the metabolism of gamma-aminobutyrate

12.2.1. Succinic semialdehyde dehydrogenase deficiency	*ALDH5A1*
12.2.2. GABA transaminase deficiency	*ABAT*

12.3. Other disorders of neurotransmitter metabolism

12.3.1. Dopamine transporter deficiency syndrome	*SLC6A3*
12.3.1. Brain Dopamine–Serotonin Vesicular Transport Disease	*SLC18A2*

13. Disorders in the metabolism of vitamins and (non-protein) cofactors

13.1. Disorders of folate metabolism and transport

13.1.1. Hereditary folate malabsorption	*SLC46A1*
13.1.2. Cerebral folate deficiency due to FOLR1 deficiency	*FOLR1*
13.1.3. Dihydrofolate reductase deficiency	*DHFR*
13.1.4. Methylenetetrahydrofolate reductase deficiency	*MTHFR*

13.2. Disorders of cobalamin absorption, transport and metabolism

13.2.1. Intrinsic factor deficiency	*GIF*
13.2.2. Enterocyte intrinsic factor receptor deficiency	
13.2.2.1. Intrinsic factor receptor deficiency due to *CUBN* mutations	*CUBN*
13.2.2.2. Intrinsic factor receptor deficiency due to *AMN* mutations	*AMN*
13.2.3. Haptocorrin deficiency	*TCN1*
13.2.4. Transcobalamin II deficiency	*TCN2*
13.2.5. Defect in adenosylcobalamin synthesis-cbl A	*MMAA*
13.2.6. Defect in adenosylcobalamin synthesis-cbl B	*MMAB*
13.2.7. Combined defect in adenosylcobalamin and methylcobalamin synthesis-cblC	*MMACHC*
13.2.8. Defect in adenosylcobalamin and/or methylcobalamin synthesis-cblD	*MMADHC*

13.2.9. Combined defect in adenosylcobalamin and methylcobalamin synthesis-cblF	*LMBRD1*
13.2.10. Transcobalamin receptor (TCblR/CD320) defect	*CD320*
13.2.10. cbl-J	*ABCD4*

13.3. Disorders of pterin metabolism

13.3.1. Guanosine 5 triphosphate cyclohydrolase I deficiency	*GCH1*
13.3.2. 6-Pyruvoyl-tetrahydropterin synthase deficiency	*PTS*
13.3.3. Sepiapterin reductase deficiency	*SPR*
13.3.4. Quinoid dihydropteridine reductase deficiency	*QDPR*
13.3.5. Pterin 4 carbinolamine dehydratase deficiency	*PCBD1*

13.4. Disorders of vitamin D metabolism and transport

13.5. Disorders of biotin metabolism

13.5.1. Biotinidase deficiency	*BTD*
13.5.2. Holocarboxylase synthetase deficiency	*HLCS*

13.6. Disorders of pyridoxine metabolism

13.6.1. Pyridoxine-dependent seizures	*ALDH7A1*
13.6.2. Pyridoxamine 5´-oxidase deficiency	*PNPO*
13.6.3. Hypophosphatasia	*ALPL*
13.6.3. Pryridoxal kinase deficiency	*PDXK*

13.7. Disorders of thiamine metabolism

13.7.1. Thiamine-responsive megaloblastic anemia syndrome	*SLC19A2*
13.7.2. Biotin-responsive basal ganglia disease	*SLC19A3*
13.7.3. Microcephaly, Amish type	*SLC25A19*

13.8. Disorders of molybdenum cofactor metabolism

13.8.1. Molybdenum cofactor deficiency	
13.8.1.1. Mo cofactor deficiency, complementation group A	*MOCS1*
13.8.1.2. Mo cofactor deficiency, complementation group B	*MOCS2*
13.8.1.3. Mo cofactor deficiency, complementation group C	*GPHN*

13.9. Other disorders of vitamins and cofactors

13.9.1. TTP1 deficiency	*TTPA*
13.9.2. Vitamin K epoxide reductase deficiency	*VKORC1*
13.9.3. Retinol binding protein deficiency	*RBP4*
13.9.4. Pantothenate kinases deficiency	*PANK2*

13.10. Disorders of riboflavin transport and metabolism

13.10.1. Riboflavin transporter deficiency	*SLC25A1*
13.10.1. Riboflavin transporter deficiency	*SLC25A2*
13.10.1. Riboflavin transporter deficiency	*SLC25A3*

14. Disorders in the metabolism of trace elements and metals

14.1. Disorder of copper metabolism

14.1.1. Menkes syndrome	*ATP7A*
14.1.1.1. Occipital horn syndrome	
14.1.2. Wilson disease	*ATP7B*

14.2. Disorder of iron metabolism

14.2.1. Hereditary haemochromatosis	
14.2.1.1. Hereditary haemochromatosis Type 1	*HFE*
14.2.1.2. Hereditary haemochromatosis Type 2	*HFE2*
	HAMP
14.2.1.3. Hereditary haemochromatosis Type 3	*TFR2*
14.2.1.4. Hereditary haemochromatosis Type 4	*SLC40A1*

14.2.2. Acoeruloplasminaemia — *CP*

14.2.3. Neurodegeneration with brain iron accumulation (NBIA) — *PANK2*, *PLA2G6*, *C19orf12*, *FA2H*, *WDR45*, *ATP13A2*

14.3. Disorder of zinc metabolism

14.3.1. Acrodermatitis enteropathica — *SLC39A4*

14.3.2. Hyperzincemia and hypercalprotectinemia

14.4. Disorder of phosphate, calcium and vitamin D metabolism

14.5. Disorder of magnesium metabolism

14.5.1. Hypermagnesaemia

14.5.1.1. Hypermanganesemia with dystonia, polycythemia, and cirrhosis — *SLC30A10*

14.5.2. Primary hypomagnesaemia

14.5.2.1. Hypomagnesaemia type 1, intestinal — *TRPM6*

14.5.2.2. Hypomagnesaemia type 2, renal — *FXYD2*

14.5.2.3. Hypomagnesaemia type 3, renal — *CLDN16*

14.5.2.4. Hypomagnesaemia type 4, renal — *EGF*

14.5.2.5. Hypomagnesaemia type 5, renal with ocular involvement — *CLDN19*

14.5.2.6. Hypomagnesaemia type 6, renal — *CNNM2*

14.5.2.7. Gitelman syndrome — *SLC12A3*

14.5.3. Secondary hypomagnesaemia

14.5.4. Hypomagnesaemic tetany

14.5.5. Hypomagnesaemia with cerebellar atrophy, hypotonia, strabismus, developmental delay, short stature, mild skeletal dysplasia, and connective tissue abnormalities — *SLC39A8*

14.6. Disorders in the metabolism of other trace elements and metals

15. Disorders and variants in the metabolism of xenobiotics

15.1. Disorders and variants of cytochrome P450-mediated oxidation

15.2. Disorders and variants of other enzymes that oxidise xenobiotics

15.2.1. Trimethylaminuria — *FMO3*

15.2.2. Dimethylglycinuria — *DMGDH*

15.3. Disorders and variants of xenobiotics conjugation

15.4. Disorders and variants of xenobiotics transport

16. Other disorders

16.1. Infantile striatal necrosis — *NUP62*

16.2. Myoclonic epilepsy of Unverricht and Lundborg — *CSTB*

16.3. Myoclonic epilepsy of Lafora — *EPM2A*, *NHLRC1*

16.4. Succinyl-CoA synthetase deficiency — *SUCLG2*

16.5. ARC Syndrome — *VPS33B*, *VIPAS39*

16.6. Sedoheptulokinase deficiency — *SHPK*

16.7. Trichohepatoenteric syndrome 1 — *TTC37*

16.8. Trichohepatoenteric syndrome 2 — *SKIV2L*

16.8. Acute necrotizing encephalopathy — *RANBP2*

List of unique genes

AARS2	*ALDOB*	*BCAT2*	*COX7B*	*EPM2A*	*GLA*	*HYAL1*	*MMACHC*	*NFU1*
AASS	*ALG1*	*BCKDHA*	*CP*	*ETFA*	*GLB1*	*IARS2*	*MMADHC*	*NHLRC1*
ABAT	*ALG11*	*BCKDHB*	*CPOX*	*ETFB*	*GLDC*	*IDH2*	*MOCS1*	*NPC1*
ABCA1	*ALG12*	*BCS1L*	*CPS1*	*ETFDH*	*GLRX5*	*IDS*	*MOCS2*	*NPC2*
ABCB11	*ALG13*	*BOLA3*	*CPT1A*	*ETHE1*	*GLS*	*IDUA*	*MOGS*	*NSDHL*
ABCB4	*ALG14*	*BTD*	*CPT2*	*EXT1*	*GLUD1*	*ISCU*	*MPDU1*	*NT5C*
ABCB7	*ALG2*	*C10orf2*	*CSTB*	*EXT2*	*GLUL*	*ITPA*	*MPI*	*NT5C3*
ABCD1	*ALG3*	*C12orf65*	*CTH*	*FA2H*	*GLYCTK*	*IVD*	*MPV17*	*NUBPL*
ABCD4	*ALG6*	*C19orf12*	*CTNS*	*FAH*	*GM2A*	*KARS*	*MRPL3*	*NUP62*
ABCG2	*ALG8*	*C1GALT1C.*	*CTSA*	*FARS2*	*GNE*	*KHK*	*MRPS16*	*OAT*
ABCG5	*ALG9*	*C7orf10*	*CTSC*	*FASTKD2*	*GNMT*	*KYNU*	*MRPS22*	*OCRL*
ABCG8	*ALPL*	*CA5A*	*CTSD*	*FBP1*	*GNPAT*	*L2HGDH*	*MSMO1*	*OGDH*
ABHD12	*AMACR*	*CAT*	*CTSK*	*FECH*	*GNPTAB*	*LAMP2*	*MTFMT*	*OPA1*
ABHD5	*AMN*	*CBS*	*CUBN*	*FGFR2*	*GNPTG*	*LARGE*	*MTHFR*	*OPA3*
ACAD8	*AMPD1*	*CD320*	*CYP27A1*	*FH*	*GNS*	*LARS2*	*MTO1*	*OPLAH*
ACAD9	*AMT*	*CETP*	*CYP7A1*	*FKRP*	*GPHN*	*LBR*	*MTPAP*	*OTC*
ACADM	*AOX1*	*CHKB*	*CYP7B1*	*FKTN*	*GRHPR*	*LCAT*	*MTR*	*OXCT1*
ACADS	*APOA1*	*CHST14*	*D2HGDH*	*FMO3*	*GSS*	*LCT*	*MTRR*	*PAH*
ACADSB	*APOA5*	*CHST3*	*DARS2*	*FOLR1*	*GUSB*	*LDHA*	*MTTP*	*PANK2*
ACADVL	*APOB*	*CHST6*	*DBH*	*FOXRED1*	*GYG1*	*LDLR*	*MUT*	*PC*
ACAT1	*APOC2*	*CHSY1*	*DBT*	*FTCD*	*GYS1*	*LDLRAP1*	*MVK*	*PCBD1*
ACOX1	*APOE*	*CISD2*	*DCXR*	*FUCA1*	*GYS2*	*LFNG*	*NAGA*	*PCCA*
ACSF3	*APRT*	*CLDN16*	*DDC*	*FXN*	*HADH*	*LIAS*	*NAGLU*	*PCCB*
ACY1	*APTX*	*CLDN19*	*DGUOK*	*FXYD2*	*HADHA*	*LIPA*	*NAGS*	*PCK1*
ADA	*ARG1*	*CLN3*	*DHCR24*	*G6PC*	*HADHB*	*LIPC*	*NDUFA1*	*PCSK9*
ADAR	*ARSA*	*CLN5*	*DHCR7*	*G6PC3*	*HAL*	*LIPI*	*NDUFA10*	*PDHA1*
ADCK3	*ARSB*	*CLN6*	*DHDDS*	*GAA*	*HAMP*	*LMBRD1*	*NDUFA11*	*PDHB*
ADSL	*ASAH1*	*CLN8*	*DHFR*	*GALC*	*HARS2*	*LPIN1*	*NDUFA12*	*PDHX*
AFG3L2	*ASL*	*CLPS*	*DHODH*	*GALE*	*HCCS*	*LPL*	*NDUFA2*	*PDK1*
AGA	*ASPA*	*CNDP1*	*DHTKD1*	*GALK1*	*HEXA*	*LRPPRC*	*NDUFA9*	*PDK2*
AGK	*ASS1*	*CNNM2*	*DLAT*	*GALNS*	*HEXB*	*MAGT1*	*NDUFAF1*	*PDK3*
AGL	*ATIC*	*COA5*	*DLD*	*GALNT12*	*HFE*	*MAN1B1*	*NDUFAF2*	*PDK4*
AGPHD1	*ATP13A2*	*COG1*	*DLST*	*GALNT3*	*HFE2*	*MAN2B1*	*NDUFAF3*	*PDP1*
AGPS	*ATP5A1*	*COG4*	*DMGDH*	*GALT*	*HGD*	*MANBA*	*NDUFAF4*	*PDP2*
AGXT	*ATP5E*	*COG5*	*DNAJC19*	*GAMT*	*HGSNAT*	*MAOA*	*NDUFAF5*	*PDPR*
AGXT2L2	*ATP6V0A2*	*COG6*	*DNAJC5*	*GATM*	*HIBCH*	*MARS2*	*NDUFAF6*	*PDSS1*
AHCY	*ATP7A*	*COG7*	*DNM1L*	*GBA*	*HLCS*	*MAT1A*	*NDUFB3*	*PDSS2*
AIFM1	*ATP7B*	*COG8*	*DOLK*	*GBE1*	*HMBS*	*MCCC1*	*NDUFB9*	*PDXK*
AKR1D1	*ATP8B1*	*COQ2*	*DPAGT1*	*GCDH*	*HMGCL*	*MCCC2*	*NDUFS1*	*PEPD*
ALAD	*ATPAF2*	*COQ4*	*DPEP1*	*GCH1*	*HMGCS2*	*MCEE*	*NDUFS2*	*PEX1*
ALAS2	*ATXN7*	*COQ6*	*DPM1*	*GCLC*	*HOGA1*	*MCOLN1*	*NDUFS3*	*PEX10*
ALDH18A1	*AUH*	*COQ9*	*DPM3*	*GCSH*	*HPD*	*MFF*	*NDUFS4*	*PEX12*
ALDH3A2	*B3GALTL*	*COX10*	*DPYD*	*GFER*	*HPRT1*	*MFN2*	*NDUFS6*	*PEX13*
ALDH4A1	*B3GAT3*	*COX14*	*DPYS*	*GFM1*	*HPS1*	*MFSD8*	*NDUFS7*	*PEX14*
ALDH5A1	*B4GALT1*	*COX15*	*EARS2*	*GFPT1*	*HSD17B10*	*MGAT2*	*NDUFS8*	*PEX16*
ALDH6A1	*B4GALT7*	*COX20*	*EBP*	*GGT1*	*HSD17B4*	*MLYCD*	*NDUFV1*	*PEX19*
ALDH7A1	*BAAT*	*COX4I2*	*EGF*	*GIF*	*HSD3B7*	*MMAA*	*NDUFV2*	*PEX2*
ALDOA	*BCAT1*	*COX6B1*	*ENO3*	*GK*	*HSPD1*	*MMAB*	*NEU1*	*PEX26*

PEX3	*PYGL*	*SLC25A20*	*TFR2*
PEX5	*PYGM*	*SLC25A22*	*TH*
PEX6	*QDPR*	*SLC25A3*	*TIMM8A*
PEX7	*RANBP2*	*SLC25A38*	*TK2*
PFKM	*RARS2*	*SLC25A4*	*TMEM126A*
PGAM2	*RBP4*	*SLC27A5*	*TMEM165*
PGAP2	*RFT1*	*SLC2A1*	*TMEM70*
PGK1	*RMND1*	*SLC2A2*	*TPMT*
PGM1	*RNASEH2A*	*SLC30A10*	*TPP1*
PHGDH	*RNASEH2B*	*SLC35A1*	*TREH*
PHKA1	*RNASEH2C*	*SLC35A2*	*TREX1*
PHKA2	*RNASET2*	*SLC35C1*	*TRIM37*
PHKB	*RPIA*	*SLC35D1*	*TRMU*
PHKG2	*RRM2B*	*SLC36A2*	*TRPM6*
PHYH	*SAMHD1*	*SLC37A4*	*TSFM*
PIGA	*SAR1B*	*SLC39A4*	*TTC19*
PIGL	*SARDH*	*SLC39A8*	*TTC37*
PIGM	*SARS2*	*SLC3A1*	*TTPA*
PIGN	*SC5DL*	*SLC40A1*	*TUFM*
PIGO	*SCARB1*	*SLC46A1*	*TUSC3*
PIGV	*SCO1*	*SLC5A1*	*TYMP*
PINK1	*SCO2*	*SLC6A19*	*UGT1A1*
PLA2G6	*SCP2*	*SLC6A3*	*UMOD*
PMM2	*SDHA*	*SLC6A8*	*UMPS*
PNLIP	*SDHAF1*	*SLC7A7*	*UPB1*
PNP	*SDHAF2*	*SLC7A9*	*UQCRB*
PNPO	*SDHB*	*SMPD1*	*UQCRQ*
PNPT1	*SDHC*	*SPG7*	*UROC1*
POLG	*SDHD*	*SPR*	*UROD*
POLG2	*SEC23B*	*SPTLC1*	*UROS*
POMGNT1	*SERAC1*	*SPTLC2*	*USF1*
POMT1	*SETX*	*SRD5A3*	*VIPAS39*
POMT2	*SGSH*	*ST3GAL3*	*VKORC1*
POR	*SHPK*	*ST3GAL5*	*VPS33B*
PPM1B	*SI*	*STS*	*WDR45*
PPOX	*SKIV2L*	*SUCLA2*	*WFS1*
PPT1	*SLC12A3*	*SUCLG1*	*XDH*
PREPL	*SLC17A5*	*SUCLG2*	*YARS2*
PRKAG2	*SLC18A2*	*SUMF1*	
PRODH	*SLC19A2*	*SUOX*	
PRPS1	*SLC19A3*	*SURF1*	
PSAP	*SLC22A5*	*TACO1*	
PSAT1	*SLC25A1*	*TALDO1*	
PSPH	*SLC25A12*	*TAT*	
PTPRZ1	*SLC25A13*	*TAZ*	
PTS	*SLC25A15*	*TCN1*	
PUS1	*SLC25A19*	*TCN2*	
PYCR1	*SLC25A2*	*TDO2*	
