# Supplementary material for: Advantages and pitfalls of an extended gene panel for investigating complex neurometabolic phenotypes
Source: Brain. 2016 Sep 6;139(11):2844–54. doi: 10.1093/brain/aww221 (PMC5091046; doi:10.1093/brain/aww221)
Supplement: Supplementary Data [file aww221_supplementary_data.zip › brain-2016-00692-File005.pdf]

Supplementary Table 1: Panel validation via identification of pathogenic variants in patients with known genetic diagnoses.

Pt	Disorder	Gene	Nucleotide change	Amino acid	SIFT	PolyPhen-2	CADD score	Reference
**D1**	Arginosuccinic aciduria	ASL	c.925G>A	p.Gly309Arg	Deleterious	Benign	33.0	(Linnebank *et al.*, 2002)
			c.919-2A>G	Splicing errors	-	-	25.1	Novel
**D2**	Hurler syndrome	IDUA	c.1469T>C<br>c.1469T>C	p.Leu490Pro<br>p.Leu490Pro	Tolerated	Benign	0.317	(Bach *et al.*, 1993)
**D3**	Pyridoxine-dependent epilepsy	ALDH7A1	c.950C>T	p.Ser317Leu	Deleterious	Possibly damaging	34.0	(Mills *et al.*, 2010)
			~6kb deletion encompassing exons 14-17	~6kb deletion encompassing exons 14-17	-	-	n/a	(Mefford *et al.*, 2012)
**D4**	Pyridox(am)ine phosphate oxidase deficiency	PNPO	c.98A>T	p.Asp33Val	Deleterious	Possibly damaging	31.0	(Schmitt *et al.*, 2010)
			c.264-21Tdel21insC	Splicing errors	-	-		(Mills *et al.*, 2014)
**D5**	Bile acid synthesis defect	CYP7B1	c.1249C>T<br>c.1249C>T	p.Arg417Cys<br>p.Arg417Cys	Deleterious	Probably damaging	35.0	(Goizet *et al.*, 2009)
**D6**	Hypermanganesemia with dystonia, polycythemia, and cirrhosis	SLC30A10	c.292_402del<br>c.292_402del	p.Val198_Phe134del<br>p.Val198_Phe134del	-	-	n/a	(Tuschl *et al.*, 2012)
**D7**	Leigh syndrome due to COX deficiency	SURF1	c.312_321del10insAT	p.Leu105Ter	-	-	33.0	(Wedatilake *et al.*, 2013)
			c.751+5G>A	Splicing errors	-	-	15.35	
**D8**	Combined oxidative phosphorylation deficiency 8	AARS2	c.2033G>A	p.Arg678Gln	Tolerated	Benign	17.04	Novel
			c.1195A>C	p.Asn399His	Deleterious	Benign	11.65	Novel
**D9**	Mitochondrial DNA depletion syndrome 8A/B	RRM2B	c.165G>A	p.Met55Ile	Deleterious	Possibly damaging	29.1	Novel
			deletion exons 4 - 6	-	-	-	n/a	Novel
**D10**	Mitochondrial complex I deficiency	NDUFS2	c.875T>C	p.Met292Thr	Deleterious	Possibly damaging	26.6	(Tuppen *et al.*, 2010b)
			c.840_842del	p.Glu280-281del	-	-	10.56	Novel
**D11**	Leigh syndrome	BCS1L	c.385G>A<br>c.385G>A	p.Gly129Arg<br>p.Gly129Arg	Deleterious	Possibly damaging	27.9	(Tuppen *et al.*, 2010a)
**D12**	Thiamine metabolism dysfunction syndrome 2	SLC19A3	c.517A>G<br>c.517A>G	p.Asn173Asp<br>p.Asn173Asp	Tolerated	Probably damaging	18.23	(Fassone *et al.*, 2013)
**D13**	Folate transporter defect	SLC46A1	c.198C>A<br>c.198C>A	p.Cys66Ter<br>p.Cys66Ter	**-**	**-**	35.0	Novel

Our panel was effective in identifying 19 of the 20 disease-causing variants in 12/13 cases where its efficacy was tested. Many of the mutations identified in these 13 patients have not been reported in the literature previously. Patient D8 harboured two missense variants in *AARS2* (p.Arg678Gln and p.Asn399His). Although these variants have been detected in population screening studies (1000 Genomes minor allele frequency < 0.01), we believe their combined effect to be pathogenic in this case. Analysis of patient D9 revealed compound heterozygosity for two novel mutations in the *RRM2B* gene. The first is a missense mutation (p.Met55Ile) that is predicted deleterious and the second is a deletion of exons 4 – 6. Patient D10 was compound heterozygous for a novel 2-nucleotide deletion (p.Glu280-281del) in *NDUFS2* alongside a known pathogenic missense mutation. We also describe a homozygous stop gain mutation (p.Cys66Ter) in *SCL46A1* not reported previously in the literature in patient D13 presenting with hereditary folate malabsorption. Whilst SIFT and PolPhen-2 predicted that the effects of the majority of the sequence variants detected would be deleterious and possibly damaging, respectively this was not the case in all instances. The p.Leu490Pro sequence change in *IDUA* of patient D2 was predicted to be tolerated/benign although this variant has been reported as causative and functional studies have shown that it affects enzyme activity. In the case of patients D8 and D12, who have a mitochondrial disorder, the predictions of SIFT and PolyPhen-2 did not agree.

References

Bach G, Moskowitz SM, Tieu PT, Matynia A, Neufeld EF. Molecular analysis of Hurler syndrome in Druze and Muslim Arab patients in Israel: multiple allelic mutations of the IDUA gene in a small geographic area. Am J Hum Genet 1993; 53(2): 330-8.

Fassone E, Wedatilake Y, DeVile CJ, Chong WK, Carr LJ, Rahman S. Treatable Leigh-like encephalopathy presenting in adolescence. BMJ Case Rep 2013; 2013: 200838.

Goizet C, Boukhris A, Durr A, Beetz C, Truchetto J, Tesson C, *et al.* CYP7B1 mutations in pure and complex forms of hereditary spastic paraplegia type 5. Brain 2009; 132(Pt 6): 1589-600.

Linnebank M, Tschiedel E, Haberle J, Linnebank A, Willenbring H, Kleijer WJ, *et al.* Argininosuccinate lyase (ASL) deficiency: mutation analysis in 27 patients and a completed structure of the human ASL gene. Hum Genet 2002; 111(4-5): 350-9.

Mefford HC, Cook J, Gospe SM, Jr. Epilepsy due to 20q13.33 subtelomere deletion masquerading as pyridoxine-dependent epilepsy. Am J Med Genet A 2012; 158a(12): 3190-5.

Mills PB, Camuzeaux SS, Footitt EJ, Mills KA, Gissen P, Fisher L, *et al.* Epilepsy due to PNPO mutations: genotype, environment and treatment affect presentation and outcome. Brain 2014; 137(Pt 5): 1350-60.

Mills PB, Footitt EJ, Mills KA, Tuschl K, Aylett S, Varadkar S, *et al.* Genotypic and phenotypic spectrum of pyridoxine-dependent epilepsy (ALDH7A1 deficiency). Brain 2010; 133(Pt 7): 2148-59.

Schmitt B, Baumgartner M, Mills PB, Clayton PT, Jakobs C, Keller E, *et al.* Seizures and paroxysmal events: symptoms pointing to the diagnosis of pyridoxine-dependent epilepsy and pyridoxine phosphate oxidase deficiency. Dev Med Child Neurol 2010; 52(7): e133-42.

Tuppen HA, Fehmi J, Czermin B, Goffrini P, Meloni F, Ferrero I, *et al.* Long-term survival of neonatal mitochondrial complex III deficiency associated with a novel BCS1L gene mutation. Mol Genet Metab 2010a; 100(4): 345-8.

Tuppen HA, Hogan VE, He L, Blakely EL, Worgan L, Al-Dosary M, *et al.* The p.M292T NDUFS2 mutation causes complex I-deficient Leigh syndrome in multiple families. Brain 2010b; 133(10): 2952-63.

Tuschl K, Clayton PT, Gospe SM, Jr., Gulab S, Ibrahim S, Singhi P, *et al.* Syndrome of hepatic cirrhosis, dystonia, polycythemia, and hypermanganesemia caused by mutations in SLC30A10, a manganese transporter in man. Am J Hum Genet 2012; 90(3): 457-66.

Wedatilake Y, Brown RM, McFarland R, Yaplito-Lee J, Morris AA, Champion M, *et al.* SURF1 deficiency: a multi-centre natural history study. Orphanet J Rare Dis 2013; 8: 96.
