# Supplementary material for: Advantages and pitfalls of an extended gene panel for investigating complex neurometabolic phenotypes
Source: Brain. 2016 Sep 6;139(11):2844–54. doi: 10.1093/brain/aww221 (PMC5091046; doi:10.1093/brain/aww221)
Supplement: Supplementary Data [file aww221_supplementary_data.zip › brain-2016-00692-File007.pdf]

RESULTS

Patients where less (or more) than two pathogenic variants were identified

A novel, likely pathogenic heterozygous variant (p.Val151Met) in the *UMPS* gene was identified in patient B2 presenting with benign orotic aciduria but a second variant in this gene was not found, despite 95% of the gene having been covered at a depth of >30x. This patient, who was screened at birth because his sibling had died of hyperammonaemia, remains asymptomatic and the orotic acid levels are consistent with a heterozygous state for UMPS deficiency. In patient B3, for whom a biochemical diagnosis of carbamoyl phosphate synthetase I deficiency had been made on the basis of severely reduced carbomylphosphate synthase activity in a liver biopsy (0.15 mmol/hr/mg protein), we detected a single heterozygous missense change (c.1010A>G; p.His337Arg) in exon 10 of CPS1, which is known to be pathogenic (Aoshima *et al.*, 2001). We were unable to find a second pathogenic change within this gene; however, only 98.5% of the gene was covered with a read-depth >30X and exon 21 had regions that were only covered at a read-depth of 3x. Finally, patient B1 harboured three heterozygous variants in the *HLCS* gene; the first variant is novel and is an insertion that causes a frameshift (p.Val512CysfsTer65) and the other two are missense variants. Whilst both p.Pro709Leu and p.Val641Met are predicted to be deleterious and probably damaging by SIFT and PolyPhen-2, respectively, p.Val641Met has been described with a minor allele frequency (MAF) < 0.01 in certain populations.

Unexpected findings or findings not fully informative of the clinical/ biochemical phenotype

Patients B6 and B7 are siblings and were found to be homozygous for a novel missense mutation in the *AASS* gene (c.965G>A). This novel variant would explain the hyperlysinaemia seen in both plasma and CSF, however would not account for the presence of global developmental delay, microcephaly, hypotonia and epilepsy. In patient U2, a homozygous duplication (c.144_151dupGCTGCGGG; p.Val51GlyfsTer50) in the *DPYS* gene was identified, which could explain the purine and pyrimidine abnormalities identified in the biochemical testing. Similarly to cases B6 and B7, the remaining features (dysplastic kidneys, eczema, microcephaly and developmental delay) are probably attributable to other, as yet un-identified genetic defects.

Moreover, our panel identified patients with clinical or laboratory findings either at the milder end of the spectrum or considered unusual for the identified gene defects. Firstly, patient U1 had a muscle biopsy twice, which was deemed unremarkable apart from increased fibre size variation. However, panel sequencing identified compound heterozygous mutations in the *POMGNT1* gene (c.1539+1G>A, c.373C>G), while excluding variants in other candidate genes such as *POMT1*, *POMT2* and *ISPD*. Although uncommon, immunofluorescence staining of glycosylated α-dystroglycan showing very mild variations from the norm have previously been reported in *POMGNT1* mutation-positive patients (Clement *et al.*, 2008). Despite the unremarkable brain imaging findings, the patient's phenotype was deemed consistent with a mild end of the spectrum *POMGNT1*-related dystroglycanopathy. Additionally, patient U7 not only had a homozygous c.178C>T mutation in the *ALDOB* gene, but was also found to be homozygous for a missense mutation (p.Gly296Asp) in the *TPP1* gene. The case's clinical severity and progression is at the milder spectrum for *TPP1* mutations, which are known to cause neuronal ceroid lipofuscinosis type 2 (MIM# 204500) and autosomal recessive Spinocerebellar Ataxia type 7 (MIM# 607998).

Protein structural analysis (Supplementary Table 6)

Four of the six variants investigated were predicted to directly affect the activity of the enzyme by altering the substrate binding pocket, and two of the variants are predicted to be destabilizing mutations which affect protein stability.

#	Protein	AA change	PDB code of structure	Structural Location	Putative consequences	FoldX prediction[a]	Tolerance of AA[b]
**B2**	**UMPS**	p.Val151Met<br><br>(*hmz*)	2WNS (human)	Located in OPRTase domain of UMPS, within one strand of the central β-sheet that forms the base of substrate binding site	Val-to-Met creates a bulkier amino acid, result in steric clashes with nearby residues, and destabilize the β-sheet	Reduces stability (3.66 kcal/mol)	**Mostly Val**, but also Leu, Cys, Ile, Ala
**U7**	**TPP1**	p.Gly296Asp<br><br>(*hmz*)	3EE6 (human)	Gly296 located in the catalytic domain, within a loop connecting an α helix and β strand	Gly-to-Asp mutation creates a bulkier amino acid, result in steric clashes with nearby residues, and destabilize the overall fold	Severely reduces stability (15.74 kcal/mol)	**Mostly Gly**, Cys
**U8**	**GALE**[c]	p.Val94Met<br><br>p.Gly95Asp<br><br>(*htz*)	1HZJ (human)	Both residues form part of substrate binding pocket, Val94 contacts the GlcNac moiety	Both Val-to-Met and Gly-to-Asp mutations create bulkier amino acids, alter shape of substrate pocket, and directly impact on substrate binding	No change in stability	**Strictly Val**<br><br>**Strictly Gly**

#	Protein	AA change	PDB code of homologue	Equivalent AA in homologue[c]	Structural Location in homologue	Putative consequences	Tolerance of AA[b]
**B8**	**PEX6**<br><br>*(type II AAA ATPase)*	p.Ala912Thr<br><br>(*hmz*)	3CF1 (human p67; 36% seq identity)	Ala912 in PEX6 is Ala685 in p67 (residue conserved)	Ala685 Forms part of ATP-binding site of D2 domain; packs against the ATP ribose moiety	Ala-to-Thr mutation creates a slightly larger amino acid, alters shape of ATP pocket, causes steric clashes with ATP ligand, hence disrupting its binding	**Mostly Ala**, but also Ser, Lys
**U4**	**AFG3L2**<br>*(type I AAA ATPase)*	p.Leu356Arg<br><br>(*hmz*)	2CE7 (bacterial, FtsH; 50% seq identity)	Leu356 in AFG3L2 is Leu209 in FtsH (residue conserved)	Leu209 follows immediately after the Walker A motif; forms part of ATP-binding site; directly interacts with ATP adenine moiety	Leu-to-Arg creates a larger, charged amino acid, alters shape of ATP pocket, causes steric clashes with ATP ligand, hence disrupting its binding	**Strictly Leu**

Supplementary Table 6: 3D structural analysis of identified variants. Analysis was performed when protein structural data were available in humans (patients U2, U15 and U16) or 'close homologues' (patients U11 and U12). [a] FoldX program predicts *in silico* if a certain mutation stabilises or destabilises protein; requires known 3D structure *a priori*. [b] Possible amino acid types found at the particular position, among the top 150 known homologues of the target protein (from CONSURF server). [c] Based on amino acid sequence alignment between target protein and homologue.

REFERENCES

Aoshima T, Kajita M, Sekido Y, Kikuchi S, Yasuda I, Saheki T, *et al.* Novel mutations (H337R and 238-362del) in the CPS1 gene cause carbamoyl phosphate synthetase I deficiency. Hum Hered 2001; 52(2): 99-101.

Clement EM, Godfrey C, Tan J, Brockington M, Torelli S, Feng L, *et al.* Mild POMGnT1 mutations underlie a novel limb-girdle muscular dystrophy variant. Arch Neurol 2008; 65(1): 137-41.

and intracellular cholesterol trafficking and cause dystonia and deafness. Nat Genet 2012; 44(7): 797-802.
